# Piperidine Derivatives: Recent Advances in Synthesis and Pharmacological Applications

**DOI:** 10.3390/ijms24032937

**Published:** 2023-02-02

**Authors:** Nikita A. Frolov, Anatoly N. Vereshchagin

**Affiliations:** N. D. Zelinsky Institute of Organic Chemistry, Russian Academy of Sciences, Leninsky Prospect 47, 119991 Moscow, Russia

**Keywords:** piperidines, piperidinones, hydrogenation, cyclization, cycloaddition, annulation, amination, multicomponent reactions, biological activity, pharmacological activity

## Abstract

Piperidines are among the most important synthetic fragments for designing drugs and play a significant role in the pharmaceutical industry. Their derivatives are present in more than twenty classes of pharmaceuticals, as well as alkaloids. The current review summarizes recent scientific literature on intra- and intermolecular reactions leading to the formation of various piperidine derivatives: substituted piperidines, spiropiperidines, condensed piperidines, and piperidinones. Moreover, the pharmaceutical applications of synthetic and natural piperidines were covered, as well as the latest scientific advances in the discovery and biological evaluation of potential drugs containing piperidine moiety. This review is designed to help both novice researchers taking their first steps in this field and experienced scientists looking for suitable substrates for the synthesis of biologically active piperidines.

## 1. Introduction

Piperidine is a six-membered heterocycle including one nitrogen atom and five carbon atoms in the sp3-hybridized state. Piperidine-containing compounds represent one of the most important synthetic medicinal blocks for drugs construction, and their synthesis has long been widespread [1]. Today, it can be unequivocally stated that heterocyclic compounds play a significant part in the pharmaceutical industry, and one of the most common in their structure is the piperidine cycle. Thus, more than 7000 piperidine-related papers were published during the last five years according to Sci-Finder. Therefore, the development of fast and cost-effective methods for the synthesis of substituted piperidines is an important task of modern organic chemistry.

**Figure 1:**
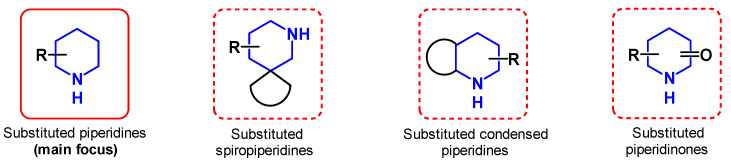
Piperidine derivatives scope of this review.

In the last several years, a lot of reviews concerning specific methods of pipiridine synthesis [2,3,4,5,6,7,8,9,10,11,12,13], functionalization [14], and their pharmacological application [15,16,17,18,19,20] were published. Herein, we have summarized the main routes in modern organic chemistry to the synthesis of piperidine derivatives (the scope is displayed in Figure 1, there and further, the atoms and bonds forming the piperidine cycle are highlighted in blue.), as well as their medical applications.

In the first chapter, approaches to the synthesis of piperidines were structured, and the features of the reaction mechanisms, the required conditions, and the limitations and advantages of particular methods were discussed. The second chapter is devoted to the main pharmacological applications of piperidine natural and synthetic derivatives, as well as recent progress in the development of new drugs of this class.

We hope that this review will provide a broad perspective on the field and will attract new creative minds to further develop the piperidine class.

## 2. Recent Advances in the Synthesis of Piperidine Derivatives

In this chapter, recent developments in the field of substituted piperidines synthesis will be discussed. It is worth noting that only examples of the piperidine ring formation will be considered and not the functionalization of already existing ones.

**Scheme 1:**
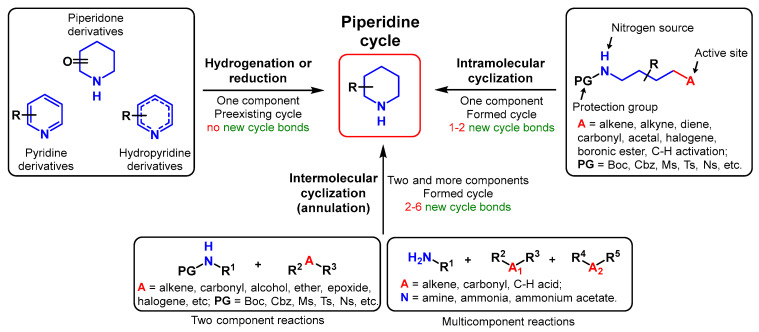
Main routes to the piperidine cycle synthesis.

For the purposes of our review, we have distinguished three main routes to the piperidines synthesis (Scheme 1).

### 2.1. Hydrogenation/Reduction

Chemists have used hydrogenation reactions since the early 19th century. This fundamental process plays a key role in modern organic synthesis. Over many decades of scientific progress, researchers have developed approaches to the hydrogenation of a wide variety of heterocyclic compounds and their derivatives, including furans [21,22,23], pyrroles [24], indoles [25], thiophenes [26], imidazoles [27], oxazolones [28], quinolines [29], etc. Pyridines are of particular interest for this review, as they are the most common source for obtaining piperidines by this method.

Now, there are many ways to achieve *N*-heteroaromatic compounds hydrogenation. Usually, the reactions take place under transition metal catalysis and harsh conditions (high temperature, great pressure, long reaction time), which makes them more expensive than the use of the other methods. Moreover, in order to meet modern pharmaceutical standards, in most cases, it is necessary to obtain a specific isomer. Thus, the reaction must be stereoselective, which is difficult in view of the aforementioned conditions. However, despite all the obvious problems of this approach, in the last decade, scientists offered various methods for overcoming them.

Here, we have discussed some of the recent developments in this field regarding the preparation of piperidines using metal- and organocatalysis. More information about *N*-heterocyclic hydrogenation methods is represented elsewhere [14,30]. 

**Scheme 2:**
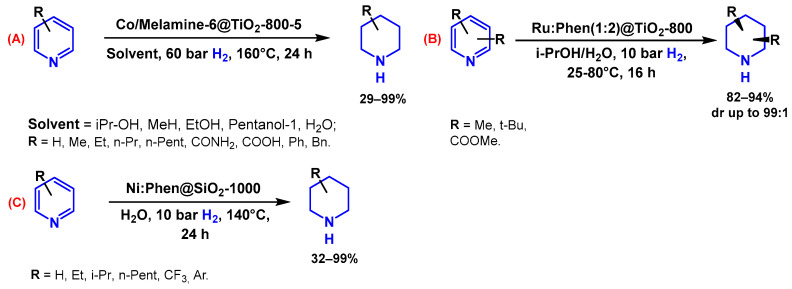
Pyridine derivatives hydrogenation using cobalt- (**A**), ruthenium- (**B**) and nickel-based (**C**) nanocatalysts.

In the work of Beller et al., a various pyridine derivatives hydrogenation was discussed [31,32,33]. A new heterogeneous cobalt catalyst based on titanium nanoparticles and melamine allowed for acid-free hydrogenation with good yields and selectivity. It was shown that it is possible to carry out the described conversions of substituted pyridines into the corresponding piperidines in water as a solvent (Scheme 2A) [31]. Moreover, the authors have optimized the method for obtaining piperidine-based biologically active substances, including Melperone, a second-generation antipsychotic [34]. Further, the Beller group developed a ruthenium heterogeneous catalyst for the diastereoselective *cis*-hydrogenation of multi-substituted pyridines (Scheme 2B) [32] and a previously unknown nickel silicide catalyst (Scheme 2C) [33]. It is the first example of a nickel catalyst’s successful application in efficient pyridine hydrogenation. It is worth noting that all catalysts possessed a high stability rate and remained effective after multiple uses.

**Scheme 3:**
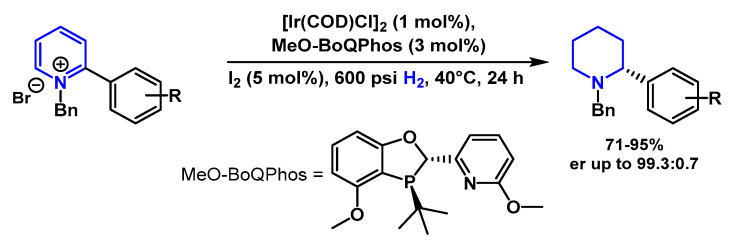
Iridium-catalyzed asymmetric hydrogenation of pyridinium salts.

Along with ruthenium and cobalt, iridium is an effective transition metal for stereoselective catalytic hydrogenation. Thus, Qu et al. reported the successful asymmetric hydrogenation of 2-substituted pyridinium salts using an iridium(I) catalyst containing a *P,N-*ligand (Scheme 3) [35]. The authors suggested that the reaction proceeds through the outer-sphere dissociative mechanism, which is known for this type of hydrogenation [36]. The reactant undergoes a series of successive protonations. The product configuration is determined by the stereoselective enamine protonation. This approach is also suitable for high-volume synthesis. Thus, the authors performed the large-scale enantioselective reduction of 2,3-disubstituted indenopyridine as part of the synthesis of a biologically active substance—11β-hydroxysteroid dehydrogenase type 1 inhibitor (11β-HSD1) [37]. 11β-HSD1 is used for treating diseases associated with cortisol abnormalities [38].

**Scheme 4:**
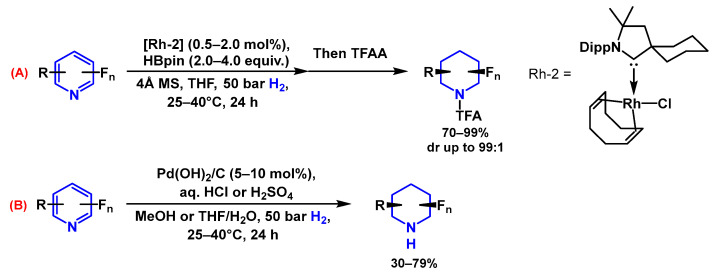
Rhodium- (**A**) and palladium-catalyzed (**B**) hydrogenation of fluorinated pyridines.

Rhodium and palladium are befitting for pyridine hydrogenation as well. For example, in 2019, Glorius et al. developed a strategy for accessing all-*cis*-(multi)fluorinated piperidines from the corresponding fluoropyridines [39,40] (Scheme 4). First, the authors used rhodium(I) complex and pinacol borane to achieve highly diastereoselective products through the dearomatization/hydrogenation process (Scheme 4A) [39]. As a result, a wide range of substituted fluoropiperidines have been obtained, including fluorinated analogs of commercially available and biologically active substances, including Melperone, Diphenidol, Dyclonine, Eperisone, and Cycrimine. The work represents a major advance in the underdeveloped field of fluoropiperidine derivatives acquirement. However, the method has its limitations in the pyridine moieties range (products with hydroxy, aryl, ester, and amide groups were not affordable) and moisture sensitivity. Therefore, in 2020, the Glorius group came up with another idea for accessing highly valuable fluorinated piperidines. The method is based on palladium-catalyzed hydrogenation (Scheme 4B) [40]. The developed approach was suitable for most substrates that were inaccessible by rhodium catalysis and was effective in the presence of air and moisture. It is worth noting that the axial-position for fluorine atoms was prevalent in the majority of experiments.

**Scheme 5:**
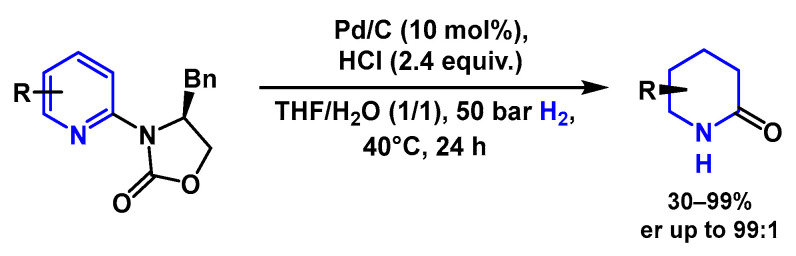
Interrupted palladium-catalyzed hydrogenation of pyridine derivatives.

The interruption of palladium-catalyzed hydrogenation by water led to piperidinones (Scheme 5) [41]. The method allows for the furthering of the one-pot functionalization of unsaturated intermediates, which usually requires multiple steps. Moreover, the reaction possessed great selectivity, high yields, and a broad substrate scope.

**Scheme 6:**
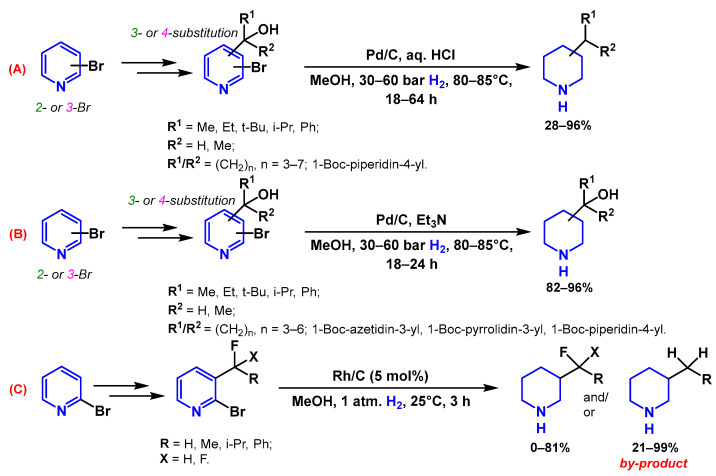
Hydrogenation of bromopyridine derivatives using palladium catalyst with hydrochloric acid (**A**) or triethylamine additive (**B**), and rhodium catalyst (**C**).

Grygorenko et al. used palladium and rhodium hydrogenation for their approach to all isomeric (cyclo)alkylpiperidines (Scheme 6) [42,43]. The method proposed by the authors combines three reactions in one step: the removal of the metalation group, dehydroxylation, and pyridine reduction (Scheme 6A). Unfortunately, due to the acid use, some substrates were not accessible. It is possible to retain the hydroxyl group with relatively higher yields by using triethylamine instead of hydrochloric acid as an additive (Scheme 6B) [42]. The rhodium catalyst proved to be more effective when 3-substituted piperidines bearing partially fluorinated groups were synthesized (Scheme 6C) [43]. The reaction took place under milder conditions and required significantly less time. Nevertheless, hydrodefluorination might occur in some cases, which leads to an undesirable by-product without any fluorine substituents.

**Scheme 7:**
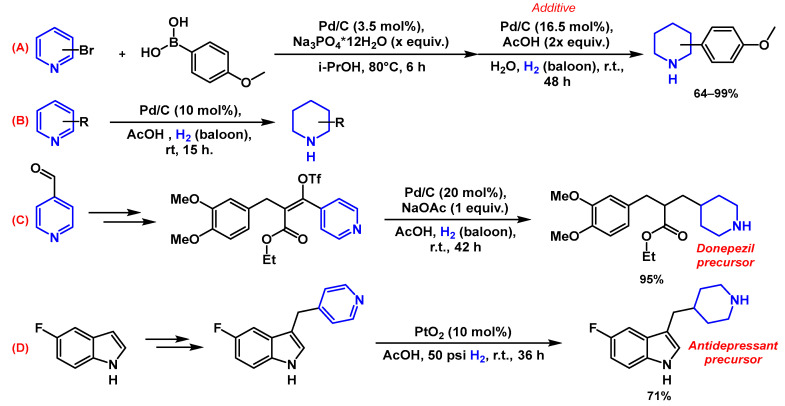
Chemoselective hydrogenation of pyridine derivatives: one-pot palladium(0)-catalyzed Suzuki–Miyaura/hydrogenation reaction (**A**), palladium(0)-catalyzed hydrogenation under ambient conditions (**B**), donepezil precursor synthesis through palladium(0)-catalyzed hydrogenation (**C**), serotonin reuptake inhibitor precursor synthesis through platinum-catalyzed hydrogenation (**D**).

Usuki et al. proposed an interesting example of functionalized chemoselective piperidine synthesis combining multiple stages in one (Scheme 7A) [44]. One-pot sequential Suzuki–Miyaura coupling and hydrogenation were carried out under mild conditions. The authors pointed out the utmost importance of maintaining the optimal starting material concentration for the successful hydrogenation process. The mild hydrogenation method was studied in detail on a broad spectrum of substrates (Scheme 7B) [45]. The authors concluded that the HOMO/LUMO states and the bulkiness of the substituents majorly influenced the reaction rate.

Moreover, chemoselectivity proved to be useful in the synthesis of donepezil (Scheme 7C), a widely used active component for Alzheimer’s disease treatment [46]. Li et al. used a similar approach in the synthesis of alkoxy-piperidine derivatives (Scheme 7D) [47]. While the indole moiety remained aromatic, the pyridine part was fully converted into piperidine.

**Scheme 8:**
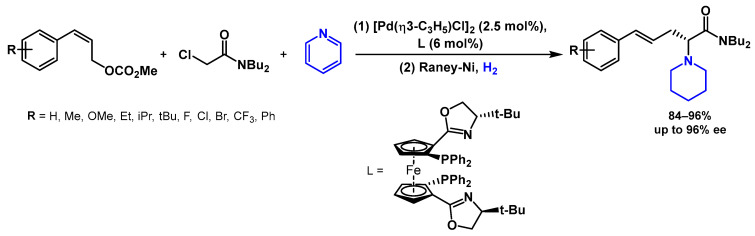
Stereoselective palladium-catalyzed cascade including pyridine hydrogenation.

Zhang et al. accomplished a stereoselective coupling/hydrogenation cascade (Scheme 8) [48]. After the coupling phase, quaternary pyridinium salt (intermediate) undergoes a partial reduction with Raney-Ni as a catalyst. If sodium tetrahydroborate is used instead of nickel, the reduction goes smoother and tetrahydropyridine is formed. The resulting piperidines can go through further transformations without enantioselectivity loss.

**Scheme 9:**
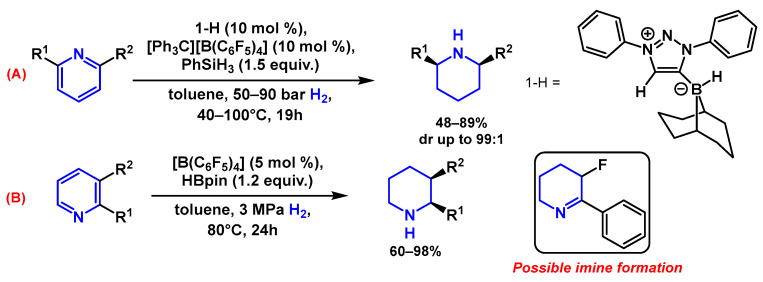
Borenium-catalyzed hydrogenation of 2,5- (**A**) and 2,3-substituted (**B**) pyridines.

Borenium and hydrosilanes are often used as a non-metal alternative in catalytic hydrogenation [5]. Thus, Crudden et al. discovered that boron ions diastereoselectively reduce substituted pyridines to piperidines in the presence of hydrosilanes (Scheme 9A) [49]. For *bis*-substituted pyridines, hydrogenation took place under mild conditions, while for *ortho*-derivatives pressure and temperature were more than doubled. Silanes are necessary to prevent the product-catalyst adduct formation. Wang et al. have developed another method for the hydroboration/hydrogenation cascade of pyridines (Scheme 9B) [50]. The method was *cis*-selective and especially effective for 2,3-disubstituted pyridines. The reaction has a number of features. For example, 2-furyl or 2-thienyl substituents undergo ring opening to form alcohols and thiols, respectively. In the reaction of 2,4-substituted pyridines, the reduction was incomplete in certain cases with the formation of tetrahydropyridines. Moreover, it is possible to obtain a cyclic imine with fluorine in the *meta* position.

**Scheme 10:**
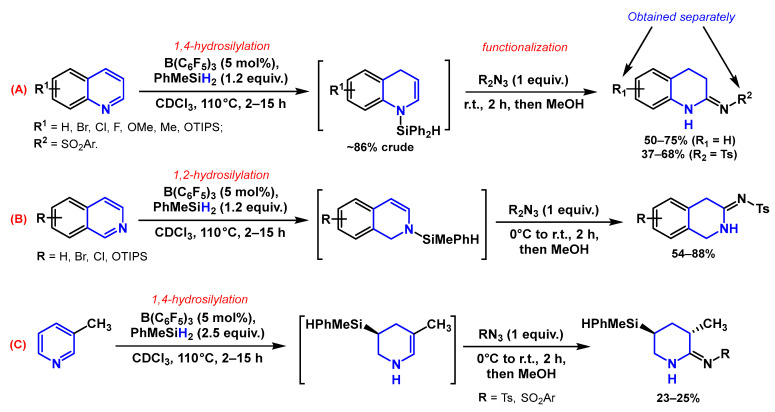
Hydrosilylation of 1- (**A**), 2-quinolines (**B**) and pyridines (**C**).

If hydrogen is not used, then, under similar conditions, as in the abovementioned work of Cruden [49], a dearomative hydrosilylation will occur. Therefore, the resulting enamine can be further functionalized. Joung et al. studied this approach in detail using the example of the hydrosilylation of quinolines (Scheme 10A) [51], isoquinolines (Scheme 10B), and pyridines (Scheme 10C) [52]. Generally, there are two variants of hydrosilylation depending on the position of the substituents in relation to the nitrogen atom. The main route is 1,4-hydrosilylation (Scheme 10A,C), when a hydride attack occurs at C4. Whenever *para*-substitution is in place, *N-*heterocycles are reduced in a 1,2-manner (Scheme 10B). Chang et al. conducted a thorough mechanistic study [53].

**Scheme 11:**
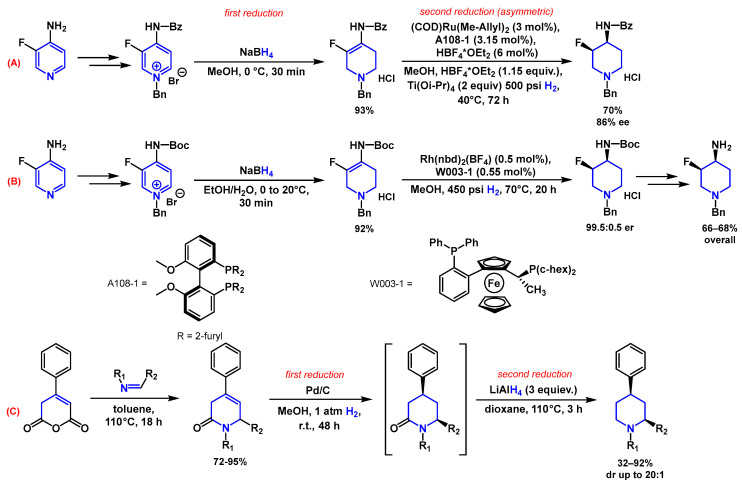
Double reduction of pyridine derivatives using ruthenium(II) (**A**) and rhodium(I) (**B**) catalyst, and piperidinone derivatives (**C**).

Double reduction is another interesting approach for the asymmetric synthesis of piperidines. Phillips et al. demonstrated the efficient asymmetric synthesis of aminofluoropiperidine as a precursor for the CGRP (calcitonin gene-related peptide receptor) antagonist (Scheme 11A) [54].

The first hydrogenation was carried out using sodium tetrahydroborate under mild conditions and with high yields. The key step was the second asymmetric hydrogenation involving a catalytic ruthenium(II) complex. Titanium isopropoxide was used to neutralize the fluorine released during the process, caused by catalyst poisoning. The procedure provides the complete conversion of enamine into the desired piperidine, with a little admixture of the defluorinated by-product (~3%). Qu et al. used a rhodium(I) catalyst with a ferrocene ligand in the similar reaction (Scheme 11B) [55]. Under these conditions, the proportion of desfluoro-impurities was less than 1%. Krasavin et al. described the stereoselective hydrogenation of unsaturated substituted piperidinones, followed by the reduction of the lactam group to give *cis*-configured 2,4-disubstituted 1-alkylpiperidines (Scheme 11C) [56].

**Scheme 12:**
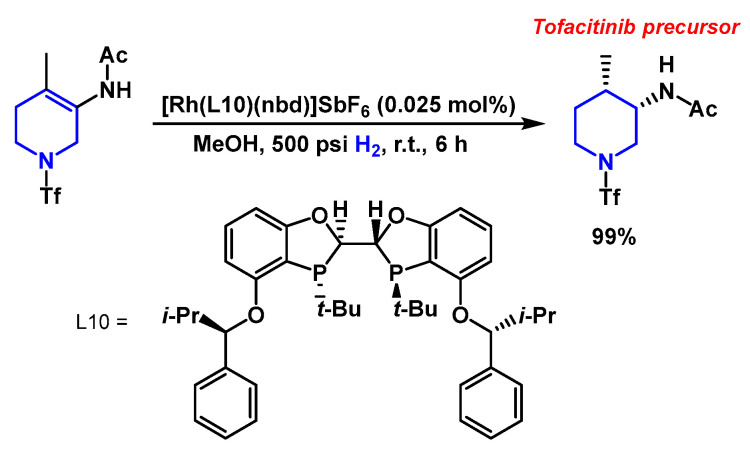
Tofacitinib precursor synthesis: rhodium-catalyzed asymmetric hydrogenation.

Previously, Li et al. proposed a catalytic complex of rhodium(I) with a *P-*chiral bisphosphorus ligand for the enantioselective asymmetric hydrogenation of aliphatic carbocyclic and heterocyclic tetrasubstituted enamides (Scheme 12) [57]. The authors showed that the interaction of the substrate and the ligands isopropyl group could play a significant role in the enantioselectivity. The developed method made it possible to carry out an efficient and practical synthesis of the Janus kinase inhibitor—Tofacitinib.

In conclusion, the synthesis of piperidines by hydrogenation/reduction has been an effective and popular approach in recent years. The main substrates are substituted pyridines with both protected and unprotected nitrogen. Most often, before hydrogenation, the substrate already has all the substituents necessary for the target product. However, current approaches combine hydrogenation and functionalization as a one-pot process, making the synthesis faster and less costly. In addition to the classic metal catalysis, the organocatalysis is gaining popularity.

### 2.2. Intramolecular Cyclization

Intramolecular cyclization (or intramolecular ring closure) is a unique case of an intramolecular reaction in which a cycle is formed within the structure of a singular molecule. Thus, the backbone of the cyclic product is entirely represented in the original reactant.

The initiation of intramolecular cyclization occurs through the activation of various functional groups or bonds. This usually requires the addition of a catalyst, an oxidizing or a reducing agent (depending on the substrate), the maintenance of the environment, etc. The main challenge for this approach is the achievement of stereo- and regioselectivity. Chiral ligands and catalysts can solve this problem. However, the selection of reaction conditions for their stability is a serious obstacle.

**Scheme 13:**
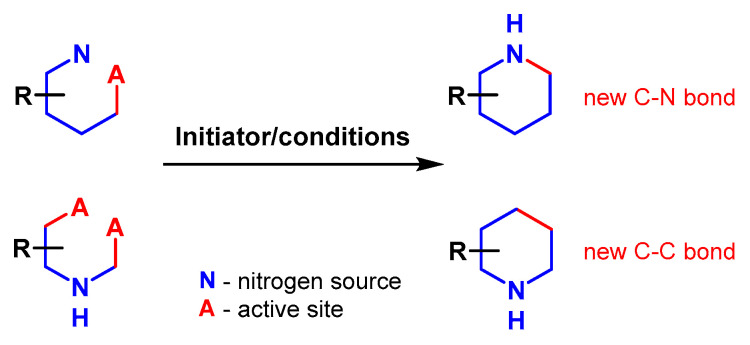
Main scheme of intramolecular cyclization.

**Figure 2:**
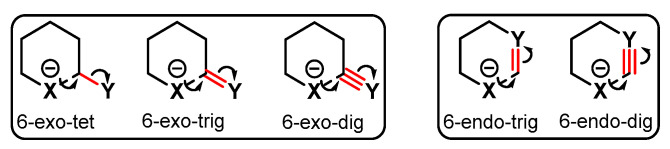
Main routes of intramolecular cyclization.

In piperidine formation by intramolecular cyclization, the substrate contains a nitrogen source (usually an amino group) and one or more active sites directly involved in cyclization. It is noteworthy that, with the direct participation of the nitrogen atom in cyclization, a new C-N bond is formed. In other cases, the formation of a new C-C bond is observed (Scheme 13). In most cases, cyclizations proceeds according to Baldwin’s rules, established by Jack Baldwin in 1976 [58] and revised by Alabugin and Gilmore in 2016 [59]. Possible variants of piperidine cyclization are shown in the figure below (Figure 2).

There are many approaches to the preparation of piperidines by intramolecular ring closure: asymmetric synthesis [60], metal-catalyzed cyclization [9,12], intramolecular silyl-Prins reaction [2,61], electrophilic cyclization [6,13], aza-Michael reaction [8], etc. Further, we outlined the recent updates on this topic within the last five years. For easier understanding, the chapter was divided by substrate classes. Reacting groups and newly formed cycle bonds are highlighted in a red color.

#### 2.2.1. Alkene Cyclization

**Scheme 14:**
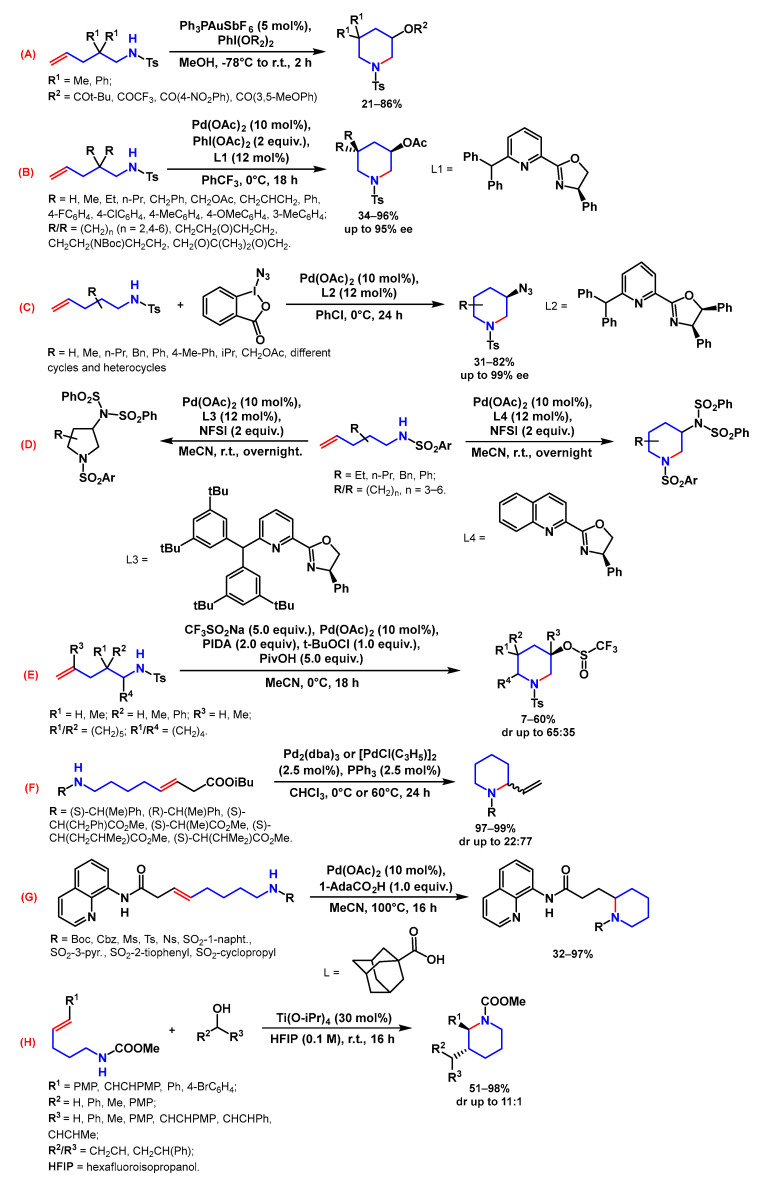
Intramolecular aminations of *N-*tethered alkenes: gold(I)-catalyzed oxidative aminoesterification (**A**), palladium(II)-catalyzed oxidative 6-endo aminoacetoxylation (**B**), palladium(II)-catalyzed azidation (**C**), palladium(II)-catalyzed 6-endo diamination (**D**), palladium(II)-catalyzed aminotrifluoromethanesulfinyloxylation (**E**), palladium(0)-catalyzed allylic amination (**F**), palladium(II)-catalyzed hydrofunctionalization (**G**), cation-induced alkylation/amination (**H**).

Nevado et al. proposed a synthetic route for the oxidative amination of non-activated alkenes to form substituted piperidines (Scheme 14A) [62]. The reaction is catalyzed by a gold(I) complex and proceeds with the use of the iodine(III) oxidizing agent. The method is designed for the difunctionalization of a double bond with the simultaneous formation of an *N-*heterocycle and the introduction of an *O-*substituent. Liu et al. developed an enantioselective approach to this reaction using a palladium catalyst (previously unknown for that type of amination) with a novel pyridine-oxazoline ligand (Scheme 14B) [63]. The authors found that a sterically bulky substituent at the C6 position of the ligand enables the palladium activation of olefins by weakening the PyN−Pd(II) bond, therefore enhancing electrophilicity. This ligand type was also effective in the azidation reaction (Scheme 14C) [64]. Moreover, Li et al. developed the ligand-controlled regioselective diamination of alkenes (Scheme 14D) [65]. In this case, a more sterically hindered ligand led to pyrrolidone formation. According to the authors, the unusual regioselectivity of the reaction arose because of a significant steric effect of the nucleophilic reagent—*N-*Fluorobenzenesulfonimide (NFSI). Shibata et al. applied palladium catalysis for the intramolecular aminotrifluoromethanesulfinyloxylation of alkenes (Scheme 14E) [66]. An intricate complex provided 6-endo-cyclized-type piperidines with moderate yields and diastereoselective ratios. Zawisza et al. represented palladium-catalyzed ligand-free diastereoselective intramolecular allylic amination (Scheme 14F) [67]. The protection group with defined stereochemistry played a crucial role in the product selectivity. Thus, the protecting group acts as a chiral ligand. Engle et al. used 8-aminoquinoline as a guide group for the palladium-catalyzed intramolecular hydroamination (Scheme 14G) [68]. The reaction is suitable for the synthesis of five and six-membered heterocycles and proceeds via *syn*-addition, a proto-depalladation mechanism. Donohoe et al. presented the stereoselective carboamination of alkenes (Scheme 14H) [69]. Cyclization is initiated by the carbocation generated in situ from alcohol. As a result, two new bonds are formed at the same time: C-C (alkylation) and C-N (carboamination).

**Scheme 15:**
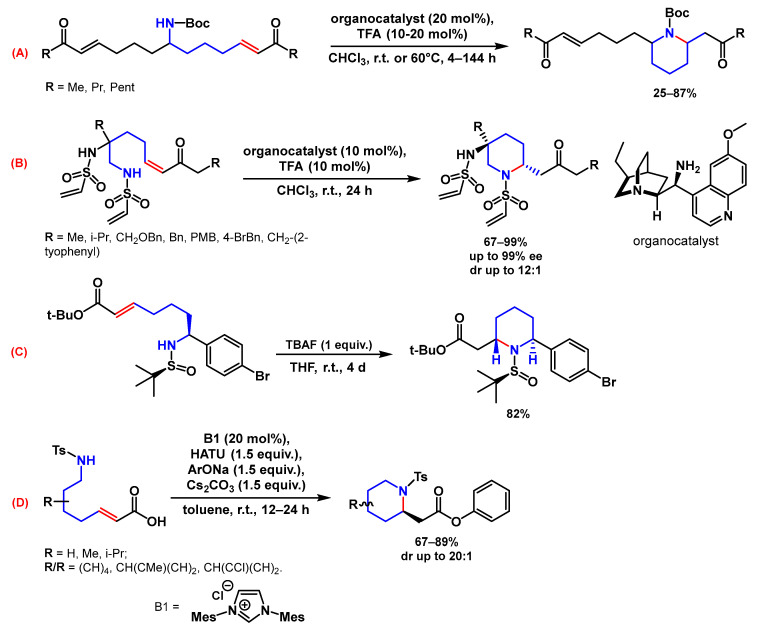
Intramolecular aza-Michael reactions of *N-*tethered alkenes: organocatalytic enantioselective synthesis of protected 2,5- (**A**) and 2,5,5-substituted piperidines (**B**), base-induced diastereoselective large-scale synthesis of 2,6-*trans*-piperidine (**C**), carbene-catalyzed diastereoselective cyclization (**D**).

Intramolecular aza-Michael reactions (IMAMR) are among the most straightforward strategies for constructing plenty of enantiomerically enriched *N-*heterocycles through double bond activation [8,70,71]. Recently, Pozo et al. proposed a general protocol for di- and tri-substituted piperidines synthesis by IMAMR using organocatalysis (Scheme 15A,B) [72,73].

The combination of a quinoline organocatalyst and trifluoroacetic acid as a cocatalyst afforded a series of enantiomerically enriched 2,5- and 2,6-disubstituted protected piperidines (Scheme 15A) and 2,5,5-trisubstituted protected piperidines (Scheme 15B) in good yields. Moreover, the authors found that the ratio of catalysts used plays a key role in the final product isomerization [72]. Bhattacharjee et al. developed the large-scale synthesis of 2,6-*trans*-piperidines through IMAMR, with TBAF as a base (Scheme 15C) [74]. For this reaction, cesium carbonate also showed good results (85% yields, *trans*/*cis* = 90/10). However, its use was difficult when scaling up due to its poor solubility. Ye et al. carried out carbene-catalyzed IMAMR (Scheme 15D) [75]. The addition of an NHC-catalyst made it possible to achieve good enantioselectivity and higher yields compared with base-only reaction.

**Scheme 16:**
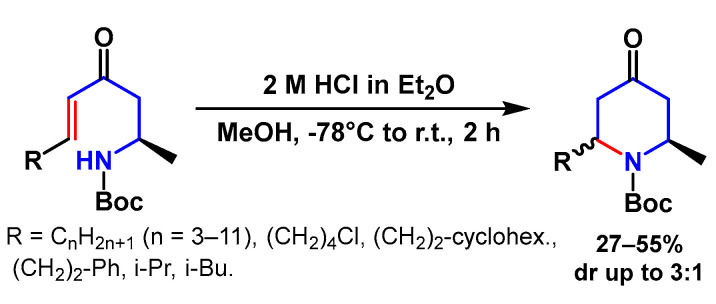
Stereoselective 6-endo-trig cyclisation.

Sutherland et al. performed a novel acid-mediated stereoselective intramolecular 6-endo-trig cyclization of enones [76] (Scheme 16). When studying the reaction mechanism, it was found that, during a long process (>2 h), the initially formed *trans*-isomer converts into a more stable *cis*-form. Therefore, a two-hour reaction was optimal for obtaining *trans*-piperidinones with a diastereomeric ratio up to 3:1.

**Scheme 17:**
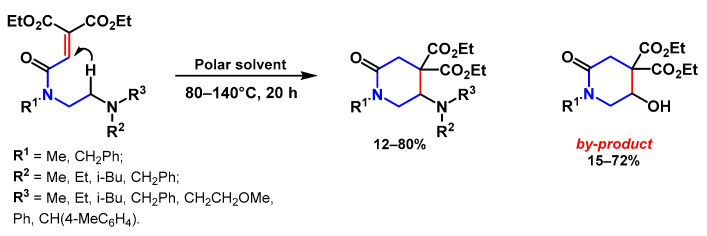
Intramolecular cyclization of alkene via hydride transfer/cyclization cascade.

Yamazaki et al. carried out the intramolecular cyclization of alkene group-bearing amides by hydride transfer [77] (Scheme 17). The formation of pipiridines with tret-amino groups proceeds efficiently in polar solvents such as DMSO, DMF, etc. However, the reaction is water-sensitive. The presence of water can lead to the loss of tertiary amino groups and the formation of a by-product with an alcohol residue.

**Scheme 18:**
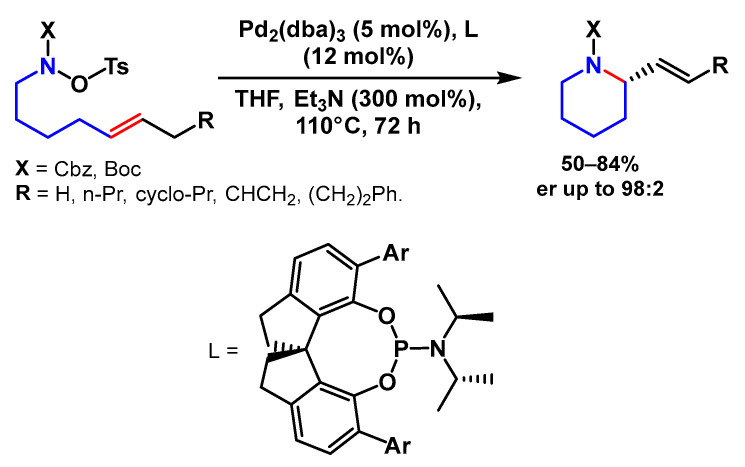
Palladium-catalyzed enantioselective 6-exo aza-heck cyclization.

Bower et al. showed the enantioselective intramolecular 6-exo aza-Heck cyclization of alkenylcarbamates [78] (Scheme 18). The main feature of the reaction is redox neutral conditions. Therefore, it can be used for the synthesis of a wide range of substrates vulnerable to oxidation. The chiral P-O ligand provides a high selectivity of the reaction. Moreover, flexible conditions and the absence of an oxidizing agent allow for the use of a palladium catalyst for further one-pot cross-coupling.

**Scheme 19:**
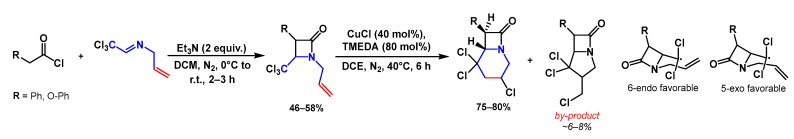
Copper(I)-catalyzed radical enantioselective cyclization.

Sadanandan and Gupta found an atypical intramolecular cyclization of β-lactams with an alkene residue [79] (Scheme 19). The reaction pathway changes from 5-exocyclization to 6-endo cyclization to form piperidine rings, contrary to Baldwin’s rule. This is due to the restraint of the double bond and the dichloromethyl radical group by the lactam ring from the formation of a convenient intermediate for 5-exo cyclization in the transition state.

**Scheme 20:**
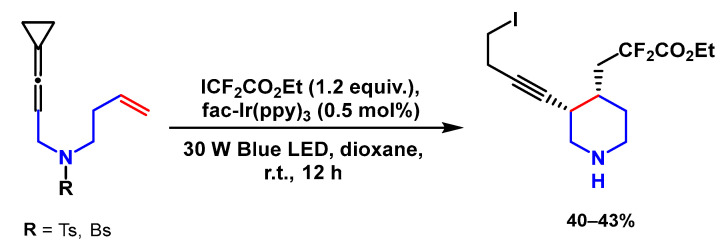
Light-mediated iridium(III)-catalyzed cyclization.

Shi et al. established a novel method of light-mediated intramolecular radical carbocyclization [80] (Scheme 20). The process was developed to obtain fluorine derivatives of heterocyclic compounds from vinylidenecyclopropanes under the action of visible light radiation.

**Scheme 21:**
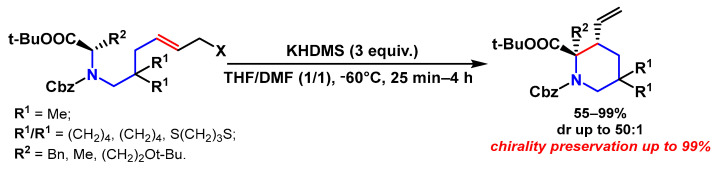
Asymmetric MOC cyclization via S_N_2-reaction.

An interesting method of alkene cyclization through the S_N_2-reaction was presented by Kim et al. [81] (Scheme 21). The chirality of the substrate was almost completely preserved. This effect is called memory of chirality (MOC). MOC was popularized by Fuji in the early 1990s and has been widely adopted ever since [82,83,84,85], including for obtaining heterocyclic compounds [86,87,88,89].

#### 2.2.2. Diene Cyclization

**Scheme 22:**
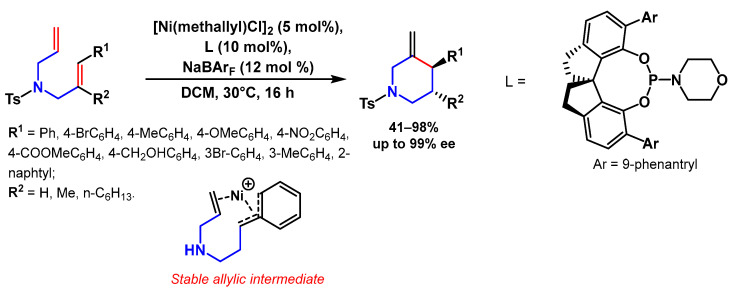
Highly enantioselective nickel-catalyzed intramolecular hydroalkenylation of 1,6-ene-dienes.

The Zhou group presented a highly enantioselective method for the intramolecular hydroalkenylation of 1,6-ene-dienes using a nickel catalyst and a chiral P-O ligand (Scheme 22) [90]. The reaction provides a regioselective mild method for the preparation of six-membered *N-* and *O-*heterocycles with an aromatic substituent and an off-cycle double bond. According to the authors’ assumptions, the nickel catalyst is coordinated on diene and then incorporated into the double bond closer to the aromatic substituent, forming a more stable allylic intermediate, which undergoes cyclization.

**Scheme 23:**
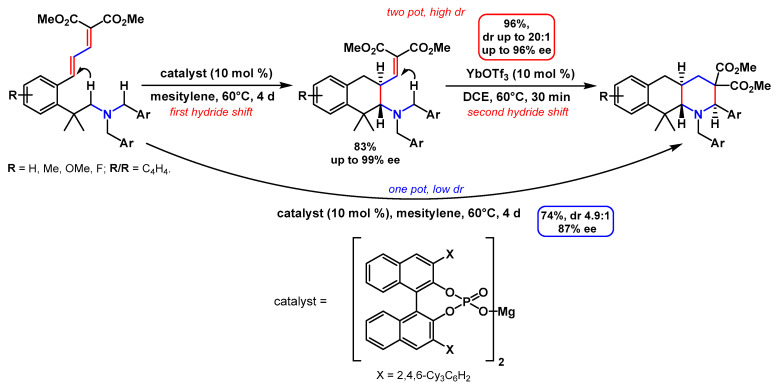
Double highly diastereoselective intramolecular cyclization of 1,3-ene-dienes via a hydride transfer/cyclization cascade.

Mori et al. proposed a method for the double C-H functionalization/cyclization of 1,3-ene-dienes with electron-withdrawing groups via a hydride shift (Scheme 23) [91]. The process is initiated by chiral magnesium biphosphate, triggering two successive 1,5-H shifts to form two cycles. The stereoselectivity of the process can be increased by using an achiral ytterbium catalyst for the second cyclization. With this approach, the diastereoselectivity increases fourfold, while high yields are maintained. The method is suitable for obtaining substituted tricyclic systems.

**Scheme 24:**
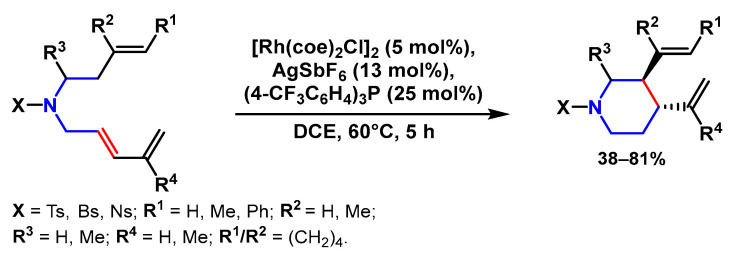
Rhodium(I)-catalyzed cycloisomerization of 1,7-ene-dienes.

The Yu group discovered the unexpected cycloisomerization of 1,7-ene-dienes (Scheme 24) [92]. After a series of experiments, the authors concluded that the length of the substrate for this reaction plays a critical role. Thus, 1,6-diens give a [4 + 2] cycloaddition product under the described conditions. The approach is excellent for obtaining a wide range of *trans*-divinylpiperidines.

**Scheme 25:**
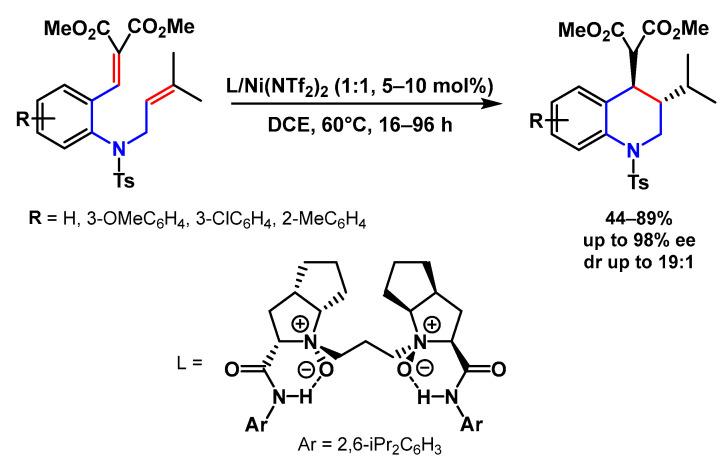
Nickel-catalyzed cyclization 1,7-ene-dienes.

Feng et al. described a new intramolecular Alder-ene reaction of 1,7-dienes using a nickel catalyst (Scheme 25) [93]. The reaction is a convenient way to obtain not only piperidines but also hydroquinoline, chromane, and thiochromane derivatives with high diastereo- and enantioselectivities. Magnesium and copper complexes are also suitable for the abovementioned method. However, nickel afforded much higher enantioselectivity values.

**Scheme 26:**
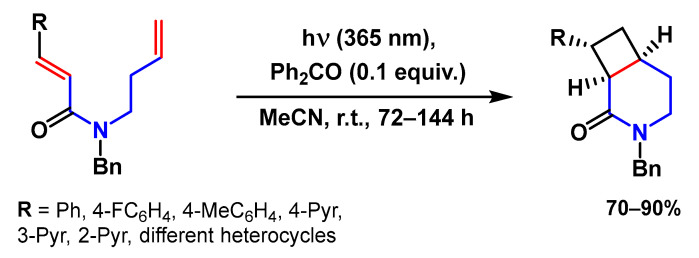
[2 + 2] Intramolecular cycloaddition of 1,7-ene-dienes.

Mykhailiuk et al. proposed a photochemical method for obtaining bicyclic piperidinones from dienes via [2 + 2] intramolecular cycloaddition [94] (Scheme 26). The resulting moieties can be easily converted into piperidines by reduction. Moreover, the reaction is scalable and proved to be useful for the synthesis of a key component analog of the antischizophrenia agent Belaperidone.

#### 2.2.3. Alkyne Cyclization

**Scheme 27:**
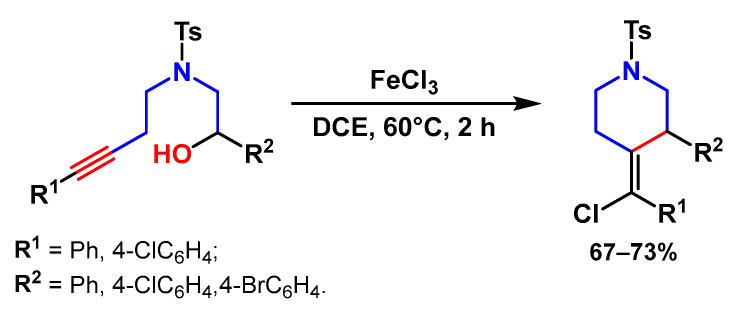
Carbenium ion-induced intramolecular cyclization.

Saikia et al. proposed a carbenium ion-induced cyclization of alkynes by the action of ferric chloride, which played a dual role as Lewis acid as well as nucleophile (Scheme 27) [95]. The method is suitable for obtaining *N-*heterocycles with alkylidene moieties. Of particular note, the reaction was E-selective for piperidines and Z-selective for pyrrolidines. The authors suggest that stereoselectivity depends on the attack of the chloride ion.

**Scheme 28:**
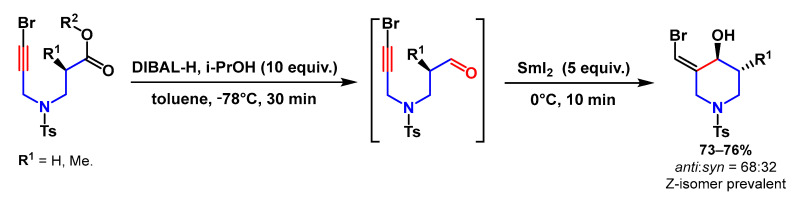
Samarium-catalyzed intramolecular radical cyclization of haloalkynals.

Takahashi et al. developed the radical cyclization of haloalkynes to produce five- and six-membered *N-*containing heterocycles [96] (Scheme 28). A halogen at the triple bond affects the reactivity of the substrate and the stereoselectivity. Thus, an E/Z ratio of 5:1 was observed when using a phenyl substituent instead of a halogen.

**Scheme 29:**
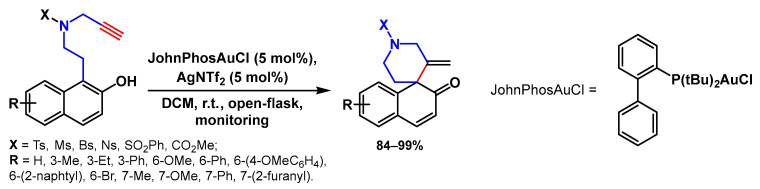
Gold(I)-catalyzed intramolecular dearomatization/cyclization.

You et al. achieved a gold-catalyzed intramolecular cyclization/dearomatization of β-naphthol derivatives with a terminal alkyne group [97] (Scheme 29). An in situ-generated gold(I) complex activates the triple bond with further 6-exo-dig cyclization and protodemetalation to afford the spironaphthalenone product. It is interesting to note that the authors did not observe the expected competitive process—*O-*cyclization.

**Scheme 30:**
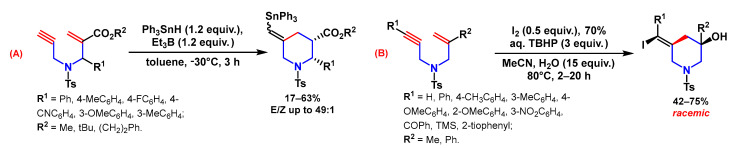
Radical stereoselective cyclization of 1,6-enynes initiated through borane addition (**A**) and oxidation (**B**).

Kamimura et al. developed a new method for the synthesis of polysubstituted alkylidene piperidines from 1,6-enynes via intramolecular radical cyclization (Scheme 30A) [98]. Triethylborane served as a radical initiator. The authors suggest the presence of a complex radical cascade, including two successive cyclizations (5-exo-dig and 3-exo-trig), cyclopropane cleavage to form a six-membered ring, and *cis*-selective hydrogen abstraction. Wang et al. used a similar approach to acquire iodo-homoallylic alcohols bearing piperidine rings (Scheme 30B) [99]. The cyclization followed the 6-endo-trig pathway using acetonitrile as the solvent and the 5-exo-trig using methanol to afford the piperidine and azobicyclic (pyrrolidone/cyclopropane) derivatives, respectively. Therefore, the reaction is regioselective. However, all the resulting products are obtained as racemates.

**Scheme 31:**
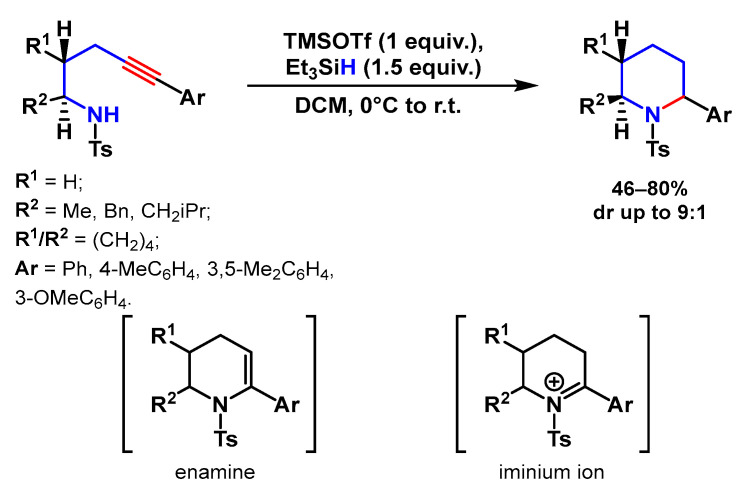
Stereoselective reductive hydroamination.

Gharpure et al. described piperidine synthesis through the intramolecular 6-endo-dig reductive hydroamination/cyclization cascade of alkynes (Scheme 31) [100]. The reaction proceeds via acid-mediated alkyne functionalization with enamine formation, which generates iminium ion. Subsequent reduction leads to piperidine formation. However, strong electron-releasing substituents (such as 4-OMe) at the aryl ring gave hydrolyzed derivatives instead of desired piperidines, while an electron withdrawing NO2 did not participate in the reaction at all.

Zeni et al. outline more information about the synthesis of six-membered *N-*heterocycles from alkynes in a big review [10].

#### 2.2.4. Radical-Mediated Amine Cyclization

**Scheme 32:**
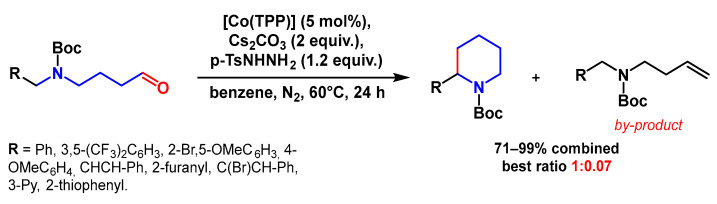
Cobalt-catalyzed cyclization.

Bruin et al. developed a new radical intramolecular cyclization of linear amino-aldehydes using a cobalt(II) catalyst [101] (Scheme 32). The reaction proceeds in good yields and is effective for the production of various piperidines and pyrrolidones. However, during the synthesis of piperidines, the appearance of a by-product in the form of the corresponding linear alkene is observed. The authors suggest the presence of a competitive process between the radical rebound and 1,5-H-transfer, which results in the formation of a by-product.

**Scheme 33:**
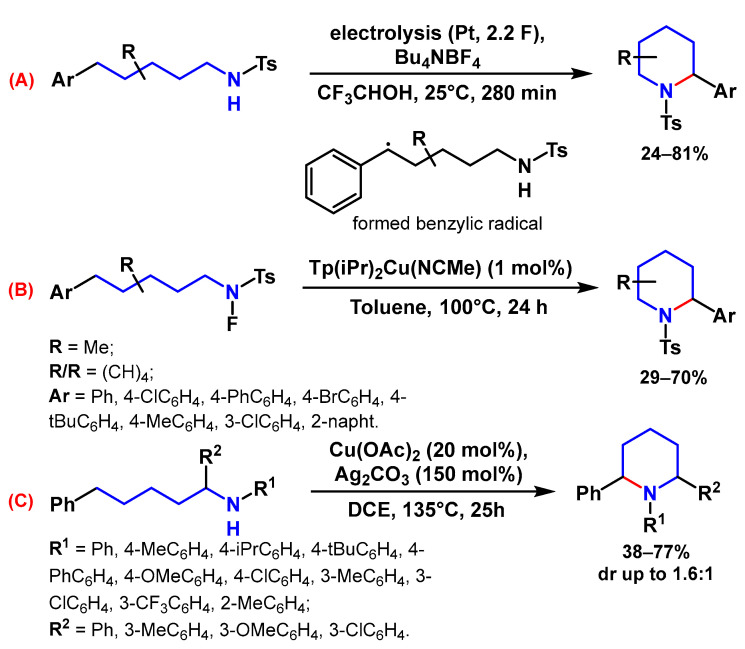
Intramolecular radical C–H amination/cyclization through electrolysis (**A**), copper(I) (**B**) and copper(II) catalysis (**C**).

Muñiz et al. developed two variants of the intramolecular radical CH-amination/cyclization of linear amines with electrophilic (aromatic) groups: anodic C-H bond activation through electrolysis (Scheme 33A) [102] and both N-F and C-H bond activation through copper catalysis (Scheme 33B) [103]. In the first variant, due to the single electron transfer, a radical cation is formed, which subsequently transforms into a benzyl radical after deprotonation. Further electron transfer results in a benzyl cation that reacts rapidly with tosylamide to give a heterocycle. The second method includes substrate coordination on a copper catalyst and further N-F bond cleavage via a single electron transfer with *N-*radical formation (N-F activation). Then, C-H activation occurs via fluorine-assisted hydrogen atom transfer, benzylic radical formation, etc. Wang et al. performed a similar approach [104] (Scheme 33C). The developed method of radical cyclization makes it possible to obtain piperidines via 1,6-hydrogen atom transfer.

**Scheme 34:**
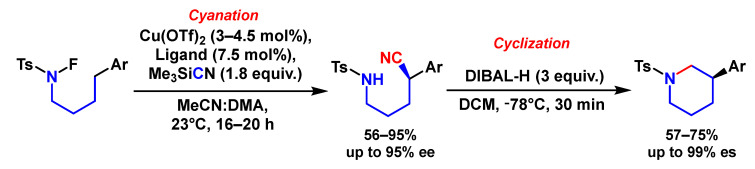
Way to piperidines through radical-mediated C-H cyanation.

Another example of the *N-*radical approach to piperidines was represented by Nagib et al. (Scheme 34) [105]. First, the enantioselective cyanidation of fluorosubstitued amines was carried out using a chiral copper(II) catalyst. The resulting aminonitriles underwent cyclization to chiral piperidines in the presence of DIBAL-H. The further optimization of the conditions made it possible to carry out the first asymmetric synthesis of an anticancer drug, Niraparib.

#### 2.2.5. Other Cyclizations

**Scheme 35:**
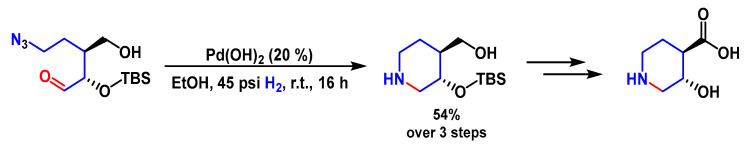
Palladium-catalyzed azide reduction cyclization.

Line accomplished the enantioselective multistage synthesis of (3S, 4R)-3-hydroxypiperidine-4-carboxylic acid, including the key one-pot azide reductive cyclization of aldehyde (Scheme 35) [106]. The resulting intermediate can be used for further modification and for obtaining various analogs of the final product.

**Scheme 36:**
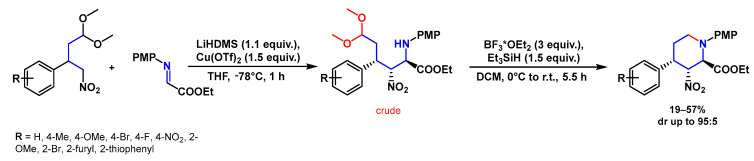
Asymmetric synthesis of piperidines by nitro-Mannich/reduction cyclization.

Anderson et al. carried out the diastereoselective reductive cyclization of amino acetals prepared by the nitro-Mannich reaction (Scheme 36) [107]. The diastereoselective Mannich reaction (first step) between functionalized acetals and imines is used to control the stereochemistry of piperidines, which is retained during reductive cyclization (second step).

**Scheme 37:**
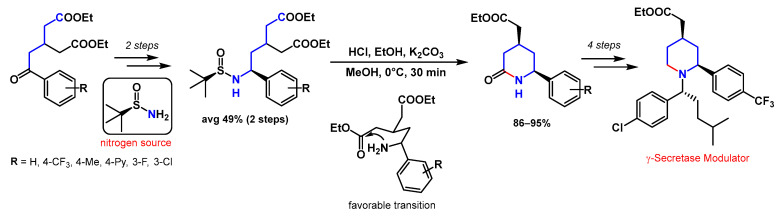
Local desymmetrization approach to piperidines.

Zu et al. developed a desymmetrization approach for piperidine synthesis through the selective lactam formation (Scheme 37) [108]. The authors extended the scope of this method for the synthesis of the γ-secretase modulator. 

**Scheme 38:**
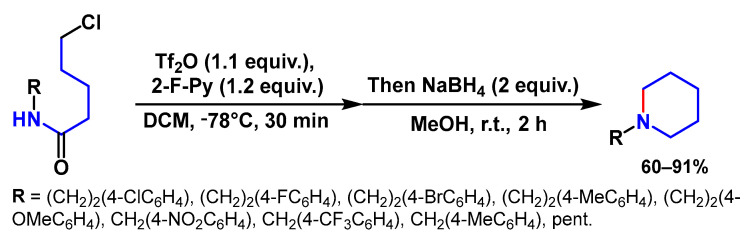
Intramolecular cyclization/reduction cascade.

Song et al. developed a one-pot cyclization/reduction cascade of halogenated amides (Scheme 38) [109]. Trifluoromethanesulfonic anhydride was used for amide substrate activation. Then, sodium tetrahydroborate was applied for imide ion reduction, followed by intramolecular nucleophilic substitution/cyclization. The reaction scope covers piperidines as well as pyrrolidines.

**Scheme 39:**
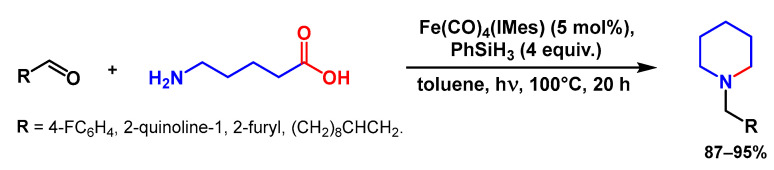
Iron-catalyzed reductive amination.

Darcel et al. developed the iron-catalyzed reductive amination of ϖ-amino fatty acids (Scheme 39) [110]. Phenylsilane plays a key role in the reaction: it promotes the formation and reduction of imine, initiates cyclization, and reduces the piperidinone intermediate with iron complex as a catalyst. The method is efficient for the preparation of pyrrolidines, piperidines, and azepanes.

**Scheme 40:**
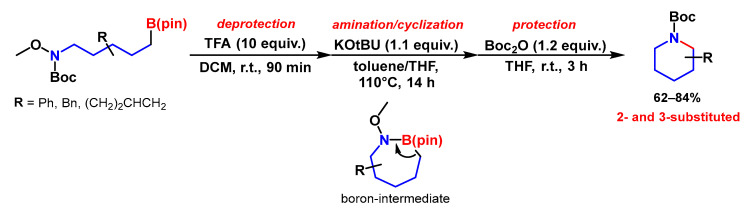
Intramolecular amination of organoboronates.

Morken et al. constructed piperidines by the intramolecular amination of methoxyamine-containing boronic esters (Scheme 40) [111]. The reaction proceeds via N-B bond formation and the 1,2-metalate shift within the boron-intermediate. Precursors can be easily obtained through the Mitsunobu reaction. Moreover, this method was optimized for boronate-containing azacycles synthesis.

### 2.3. Intermolecular Cyclization (Annulation)

The intermolecular annulation process consists in the formation of a cycle of two or more components. Therefore, it can be divided into two-component reactions and multicomponent reactions, which will be discussed further.

#### 2.3.1. Two-Component Reactions

In the two-component intermolecular preparation of piperidines, the formation of two new bonds’ combination is mainly observed: C-N and C-C or two C-N. The condensation of amines with either aldehydes or ketones and with the further reduction of the imine group, also known as reductive amination, is one of the commonly used methods of C-N bond formation. This approach was applied mostly in [5 + 1] annulations.

**Scheme 41:**
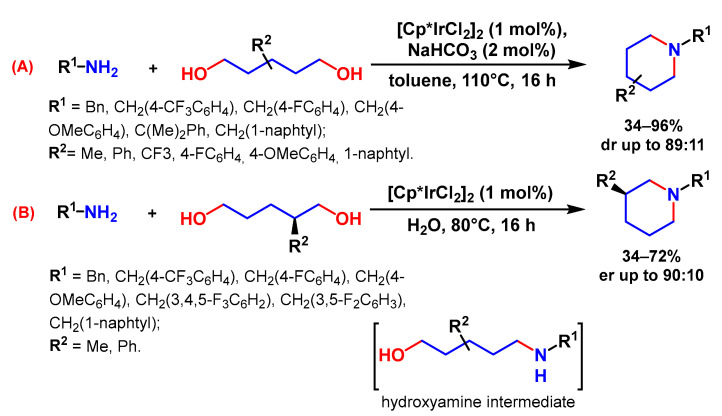
Hydrogen borrowing annulation of diols (**A**) and enentioenriched diols (**B**).

A hydrogen borrowing the [5 + 1] annulation method was reported by Donohoe et al. (Scheme 41) [112]. The mechanism includes two iridium(III)-catalyzed sequential cascades of hydroxyl oxidation, amination, and imine reduction by hydrogen transfer via a metal catalyst. The first amination is intermolecular (hydroxyamine intermediate formation), and the second is intramolecular. Thus, two new C-N bonds are formed. This approach enables the stereoselective synthesis of substituted piperidines. Moreover, the use of water as a solvent prevents the racemization of enantioenriched substrates, providing a route to highly enantioselective C4-substituted piperidines (Scheme 41B).

**Scheme 42:**
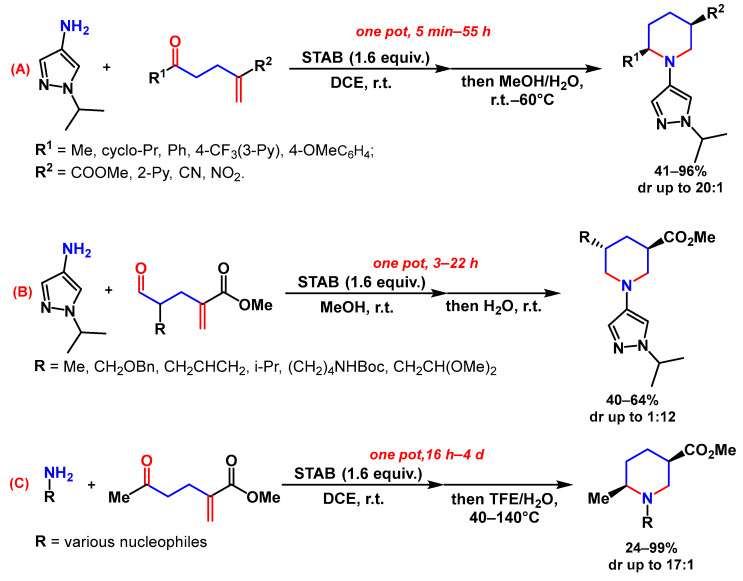
Diastereoselective [5 + 1] annulation of amines with aldehydes (**A**,**B**) and ketoesters (**C**).

Sather et al. proposed a new approach to piperidines through a combination of reductive amination and IMAMR (see Section 2.2.1) [113]. The reaction possesses predictable diastereoselectivity. Whenever ketone is used as a substrate, *trans*-selectivity is observed, and for aldehyde, the process is *cis*-selective, with a dr up to 20:1 and 1:12, respectively (Scheme 42).

**Scheme 43:**
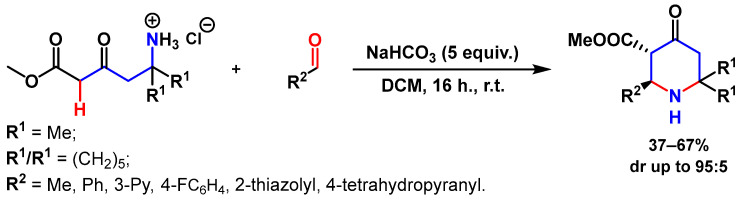
Amino-ketoester cyclization with aldehydes.

**Scheme 44:**
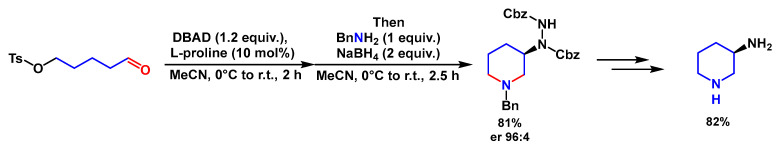
1,2-Diamination of aldehydes.

Griggs et al. further explored a stereoselective route to non-symmetrical spiropiperidinons via a similar condensation/intramolecular cyclization cascade (Scheme 43) [114]. The C-H acid center acted as an active cyclization site. The enantioselective synthesis of piperidines via the 1,2-diamination of aldehydes was reported by Ramapanicker et al. (Scheme 44) [115]. The initial alpha-amination of aldehyde with dibenzyl azodicarboxylate (DBAD) was followed by reductive amination/cyclization. This method is designed for the stereoselective synthesis of amine-substituted piperidines.

**Scheme 45:**
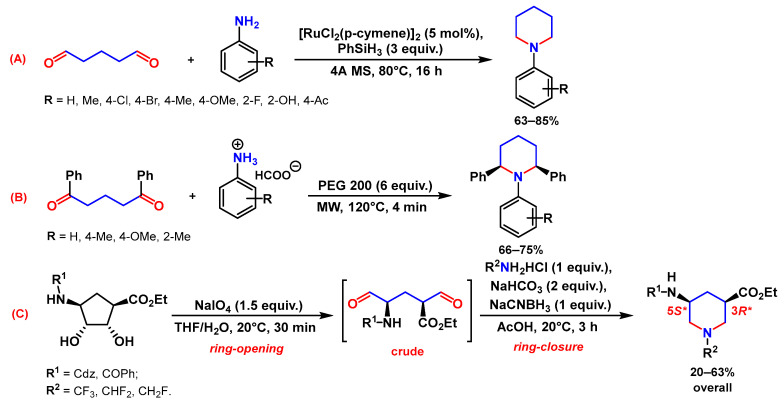
Double reductive aminations via ruthenium(II) catalysis (**A**), microwave radiation (**B**) consecutive oxidative ring opening and reductive ring closure (**C**).

Double reductive aminations are an effective route to piperidines. Thus, Jiang et al. proposed the highly selective ruthenium(II)-catalyzed double reductive amination/hydrosylilation of glutaric dialdehyde and aniline derivatives (Scheme 45A) [116]. It is worth noting that the method is suitable only for amine substrates with the p-π conjugation effect. Rao et al. achieved a similar process through a microwave-mediated Leuckart reaction (Scheme 45B) [117]. Piperidines were afforded from diketone and aryl ammonium formate, which played a double role as a nitrogen source and reductant. Kiss et al. developed the stereocontrolled synthesis of fluorine-containing piperidines from racemic cyclic diols (Scheme 45C) [118]. The chain of reactions proceeded through an oxidative ring opening to form an unstable dialdehyde, which immediately underwent double reductive amination/ring closure with a fluorine-containing quaternary ammonium salt. Sodium cyanoborohydride was used as a reducing agent.

**Scheme 46:**
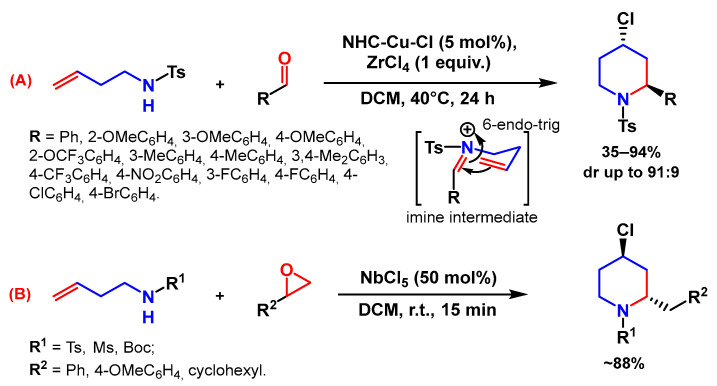
Aza-Prins cyclizations of homoallylic amines with aldehydes (**A**) and epoxides (**B**).

The aza-Prins reaction is another efficient way to accomplish piperidine synthesis [119]. Thus, Li et al. proposed the aza-Prins cyclization of homoallylic amines with aldehydes promoted by the NHC-Cu(I) complex and ZrCl_4_ (Scheme 46A) [120]. After the aldehyde group activation by zirconium chloride, an iminium intermediate was obtained; it further underwent 6-endo-trig cyclization with the formation of a carbocation in the 4-position. The *trans*-selectivity of the reaction is explained by steric hindrance. Thus, a nucleophilic attack of the chloride ion from the axial side is less favored. Rajasekhar et al. carried out a similar route with epoxides (Scheme 46B) [121]. There, niobium pentachloride served as Lewis acid and a chlorine source.

**Scheme 47:**
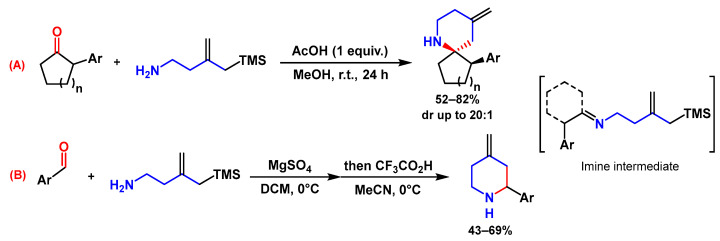
Aza-Sakurai cyclizations of allylic amines with cyclic ketones (**A**) and aldehydes (**B**).

Martin et al. developed [5 + 1] aza-Sakurai cyclization for spiropiperidines (Scheme 47A) [122] and piperidines (Scheme 47B) [123] synthesis using amines with cyclic ketones and aldehydes, respectively. The process includes an intermolecular reaction of imine formation through condensation, followed by intramolecular cyclization. The resulting piperidines carry three functional centers (NH, olefin, aromatic groups) suitable for further derivatization.

**Scheme 48:**
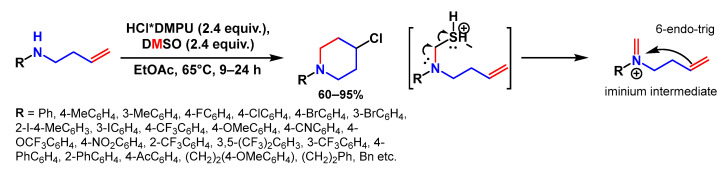
A [5 + 1] acid-mediated 4-chloropiperidines synthesis.

Xu et al. displayed a [5 + 1] acid-mediated annulation through the aza-Pummerer approach (Scheme 48) [124]. The acid complex used promotes the formation of the carbenium ion from dimethyl sulfoxide via Pummerer fragmentation. Moreover, hydrochloric acid acts as a chlorine source. Thus, three new bonds were formed: C-N, C-C and C-Cl.

**Scheme 49:**
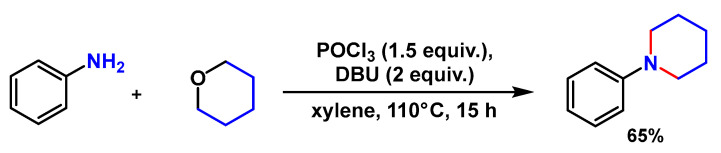
Synthesis of N-aryl-substituted azacycles from cyclic ethers.

Kim et al. proposed the metal-free *N-*heterocyclization of arylamines with cyclic ethers (Scheme 49) [125]. The reaction took place with the use of phosphoryl chloride, which initiated the ether ring-opening and the subsequent piperidine ring-closure, with two new C-N bond formations overall.

In addition to [5 + 1] annulation, [4 + 2] and [3 + 3] reactions have also been applied in piperidine synthesis.

**Scheme 50:**
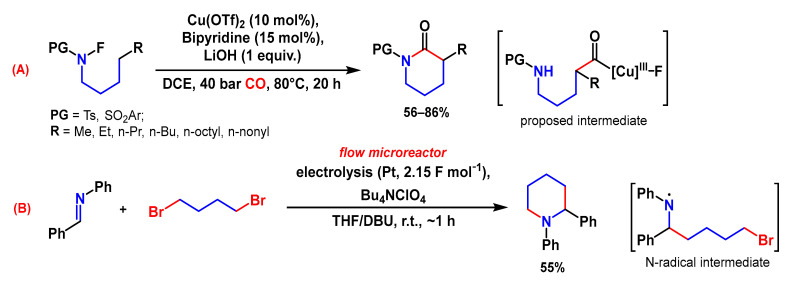
Radical-mediated intermolecular annulations through copper(II) catalysis (**A**) and electrolysis (**B**).

Wu et al. represented another variant of radical-mediated cyclization (Scheme 50A) [126]. As in the abovementioned works (see chapter 2.2.4), the copper catalyst initiates *N-*radical formation, and afterwards, the 1,5-HAT carbon radical is captured by CO and Cu(II) species to form the proposed intermediate, followed by an intramolecular ligand exchange and reductive elimination/cyclization. It is worth noting that the intermediate could undergo an intermolecular exchange with alcohols giving esters. Atobe et al. introduced a flow microreactor for the radical electroreductive cyclization of imines with terminal dihaloalkanes (Scheme 50B) [127]. The reaction goes through radical anion formation, which is involved in the intermolecular nucleophilic attack on the terminal dihaloalkane. Then, the earlier-formed *N-*radical undergoes one-electron reduction and intramolecular cyclization.

**Scheme 51:**
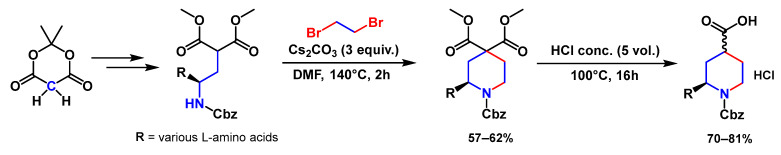
Enantiospecific synthesis of 2-substituted piperidine-4-carboxylic acids.

Balakumar et al. presented a methodology for the synthesis of 2-substituted carboxypiperidines from amino acids with a known stereochemistry (Scheme 51) [128]. The cycle was formed in the alkylation stage of the amine with dihaloalkane in high yields.

**Scheme 52:**
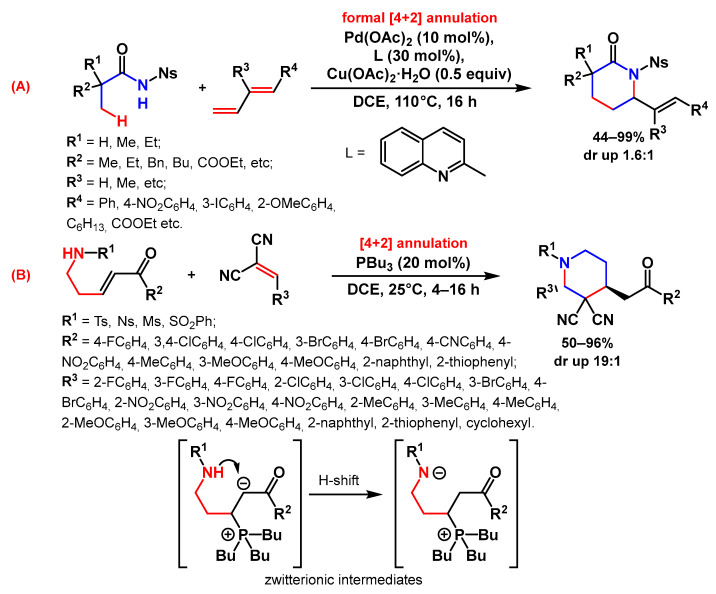
Piperidine synthesis through palladium(II)-catalyzed (**A**) and organocatalytic (**B**) [4 + 2] cycloaddition.

Gulías et al. developed a palladium that promoted the formal [4 + 2] oxidative annulation of alkyl amides and dienes (Scheme 52A) [129]. A feature of this reaction is the activation of C(sp3)-H bond, which is atypical for cycloadditions. The authors proposed that the activation and cleavage of C(sp3)-H bond were processed through migratory insertion/reductive elimination mechanisms. The diene substrate is essential to directing the process towards reductive elimination. Guo et al. showed the organocatalytic [4 + 2] annulation of *N-*tethered enones and dicyanoalkenes (Scheme 52B) [130]. The reaction tolerated a broad scope of substrates and possessed high yields and great diastereoselectivity.

**Scheme 53:**
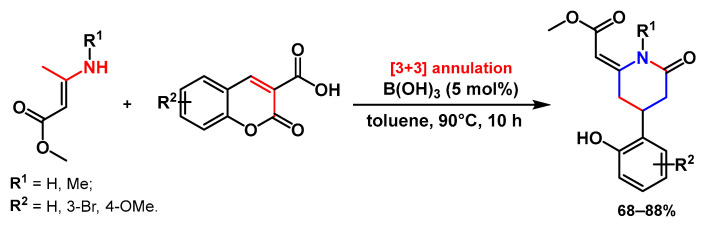
Piperidine synthesis through [3 + 3] cycloaddition.

The [3 + 3] cycloaddition method has increasingly attracted the attention of the scientific community for the synthesis of heterocyclic compounds in recent years [131,132]. Thus, Yang et al. described the regioselective [3 + 3] annulation of enones with α-substituted cinnamic acids (Scheme 53) [133]. The reaction proceeded through intermolecular Michael addition, decarboxylation, and intramolecular lactamization/cyclization.

#### 2.3.2. Multicomponent Reactions

**Scheme 54:**
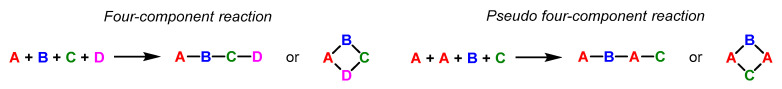
Main scheme of MCRs.

By definition, multicomponent reactions (MCRs) include one-pot processes in which three or more components interact to form a target compound containing, in its structure, the majority of atoms of all starting substances (Scheme 54) [134,135]. Reactants are mixed in one reaction vessel, without the introduction of additional reagents during the reaction process. MCRs have a number of significant advantages compared to two-component reactions: the simplicity and availability of reagents, the reduction in the number of synthesis stages, the simplification of the process of isolating final compounds, the reduction in solvent consumption, and, as a result, their environmental friendliness and higher efficiency.

Multicomponent synthesis is firmly rooted in organic chemistry as the main way to obtain various classes of compounds. MCRs are often used in the complete synthesis of complex natural compounds, require a minimum set of starting materials, and make it possible to obtain extensive libraries of compounds that have a structure similar to that of biologically active drug components.

Reactions of this type make a great contribution to the convergent synthesis of complex organic molecules, which are of great importance for the pharmaceutical industry, biochemistry, and research in the field of medicine [136].

MCRs include a variety of methods such as the Strecker synthesis, the Hanch synthesis of dihydropyridins and pyrroles, the Biginelli reaction, the Mannich reaction, the Ugi reaction, the preparation of imidazoles according to Radziszewski, the Passerini reaction, and many others [137,138,139,140,141]. MCRs have an indisputable importance in modern organic chemistry.

**Scheme 55:**
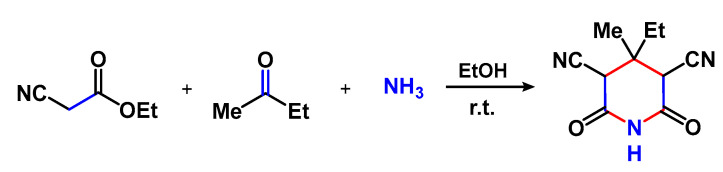
Pseudo four-component synthesis of cyclic imide.

One of the first examples of the multicomponent synthesis of compounds containing a piperidine fragment is shown in the work of Guareschi in 1897 (Scheme 55) [142]. The synthesis was carried out by an MCR between butanone, ethyl cyanoacetate, and an alcohol solution of ammonia to obtain a cyclic imide. The yield of the final imide was not reported in the work.

This work was followed by a series of publications devoted to the preparation of Guaresi imides in a multicomponent variant using an alcoholic solution of ammonia, ethyl cyanoacetate, aldehydes and ketones of various structures [143,144].

In recent years, the number of publications devoted to the multicomponent synthesis of piperidine-containing compounds is relatively small in comparison with multistage methods. However, the scientific world community still offers new approaches to solving the problems of multistage syntheses using MCRs. Further, we highlighted some of the last year’s works related to the multicomponent synthesis of various substituted piperidine cycles. It is worth noting that MCRs are more often used for obtaining piperidine cycles with double bonds (tetrahydropyridines). However, this is beyond the scope of this review. You can find more examples of this method in recently cited publications [3,145,146,147,148,149,150,151,152,153,154].

**Scheme 56:**
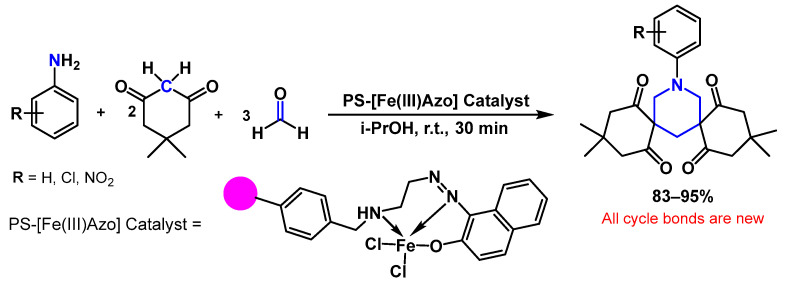
Pseudo six-component synthesis of spiropiperidine derivatives.

**Figure 3:**
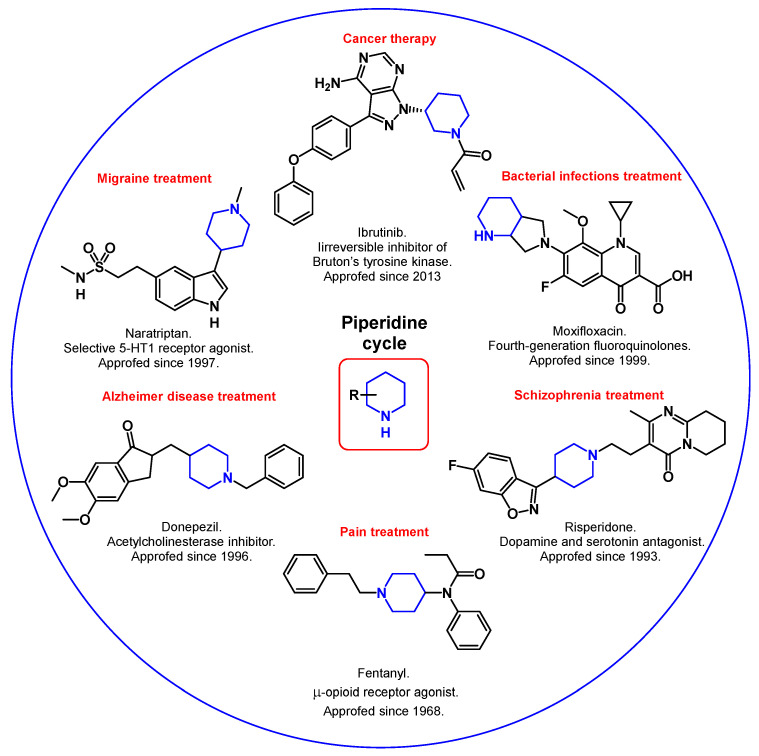
Synthetic piperidine derivatives in medicine.

Islam et al. invented a novel polystyrene ferric-based azo-catalyst for the highly effective synthesis of spiropiperidine derivatives (Scheme 56) [155]. Primary aromatic amine was used as a nitrogen source (Figure 3). The described catalyst worked much better than raw FeCl_3_*6H_2_O, allowing for high yields, the full conversion of reactants without any heating, and the recyclability of the catalyst.

**Scheme 57:**
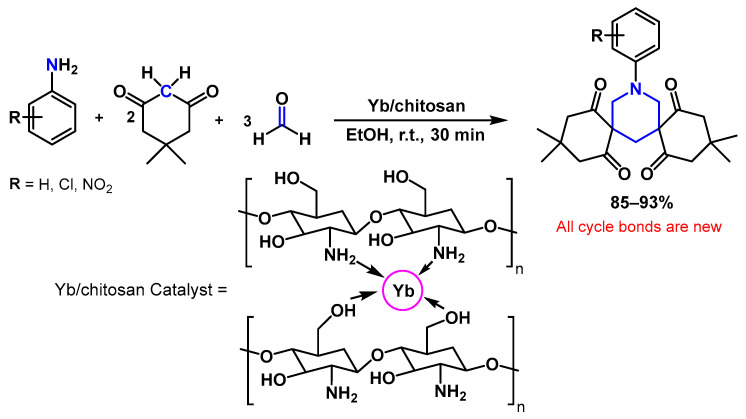
Pseudo six-component synthesis of spiropiperidine derivatives.

In the work of Ahmad et al., the obtainment of the same compounds was achieved with a chitosan-supported ytterbium heterogeneous nano-catalyst (Scheme 57) [156]. The Yb/chitosan catalyst was active with acyclic methylene compounds and dimedons in the same conditions. Thus, the method used is also suitable for the preparation of piperidines without spirocyclic substituents.

**Scheme 58:**
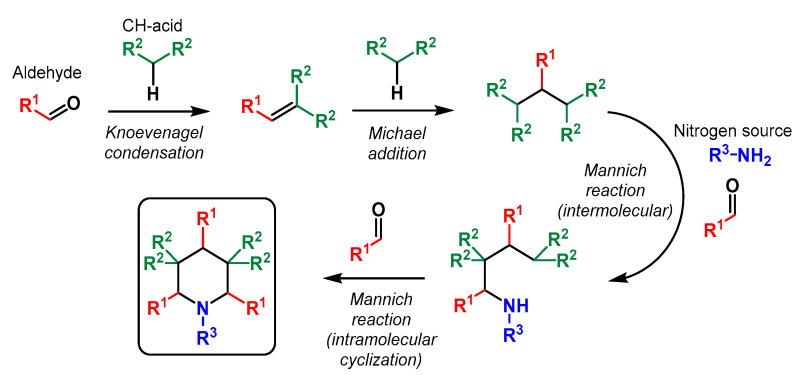
Common cascade mechanism of piperidine cycle formation.

In the described methods, piperidine cycle formation was reached through a domino process of different mechanisms including Knoevenagel condensation, Michael addition, and two consecutive Mannich reactions. Such cascade is common for most of the multicomponent synthesis of piperidines (Scheme 58). More information about MCRs combinations of «name reactions» can be found here [157].

For example, Vereshchagin’ group developed a stereoselective variant of the abovementioned approach for the synthesis of poly-substituted piperidines using ammonium acetate both as a nitrogen origin and catalyst for CH-acid deprotonation [158,159,160,161,162,163]. Ammonium acetate plays a key role in the synthesis of various compounds in organic chemistry, including its use in MCRs. Reactions with ammonium acetate are widely adopted [164]. Therefore, the preparation of γ-lactams [165], furo [3,2-c]chromen-4-ones [166], imidazoles [167,168,169,170,171], triarylpyridines [172], substituted 3-cyanopyridines [173], dihydropyridines [174,175] of various structures, and many other compounds through multicomponent processes is carried out using ammonium acetate. The ease of its use and its commercial availability make ammonium acetate one of the most common reagents utilized to introduce one or more nitrogen atoms into the structure, which is essential in multicomponent processes [176].

**Scheme 59:**
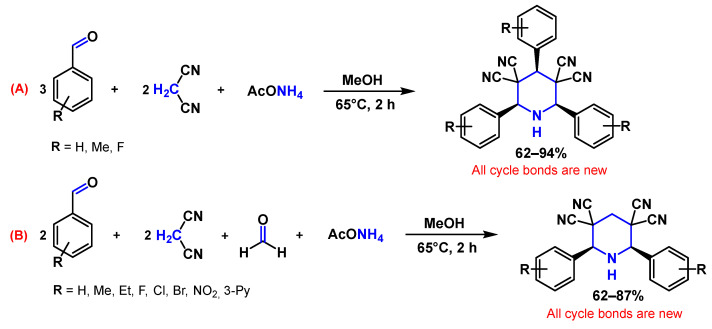
Stereoselective one-pot pseudo six-component synthesis of poly-substituted piperidines with three (**A**) and two (**B**) aromatic substituents.

First, the authors obtained a series of piperidine diastereomers with aromatic substituents from benzaldehydes, malononitrile and ammonium acetate in pseudo six-component synthesis (Scheme 59A) [158]. The reaction showed high yields and great stereoselectivity. Then, the authors added formaldehyde to the MCR system in order to achieve a product without the third aromatic ring (Scheme 59B) [159]. The researchers proposed that formaldehyde undergoes Knoevenagel condensation instead of benzaldehyde, which, in the original method, participates only in the Mannich reaction.

**Scheme 60:**
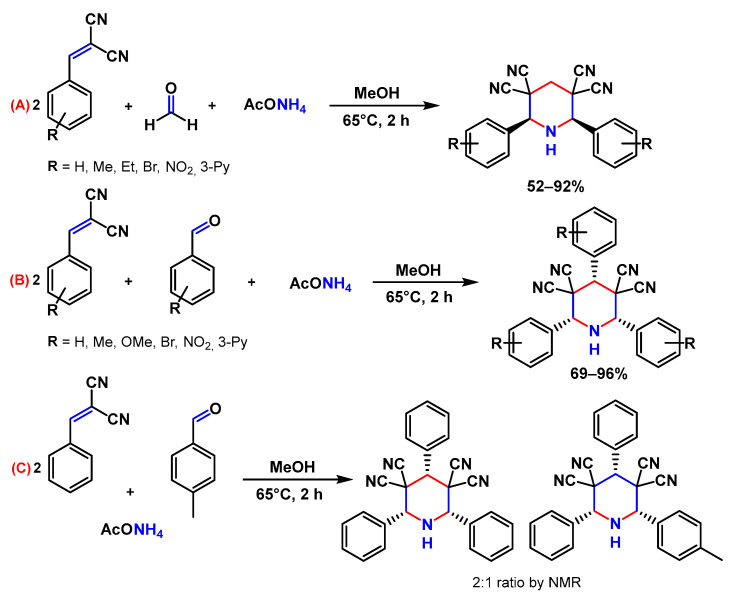
Stereoselective one-pot pseudo four-component synthesis of poly-substituted piperidines with two (**A**) and three (**B**) aromatic substituents, and example of retro-Knoevenagel condensation (**C**).

The same approach was applied when the product of Knoevenagel condensation was obtained separately (Scheme 60) [160,161]. In this case, the reaction goes through a Michael/Mannich cascade with four new bonds formed. However, this approach has several limitations. When trying to obtain piperidines with different substituents in the benzene fragments, a mixture of a two-to-one ratio is observed using ammonium acetate, and one of a three-to-one ratio is observed using aqueous ammonia (Scheme 60C) [161]. The authors suggest that the by-product is formed due to competitive mechanisms. In parallel with the Michael/Mannich cascade, retro-Knoevenagel condensation occurs with the formation of benzaldehyde, which then participates in the further transformation.

**Scheme 61:**
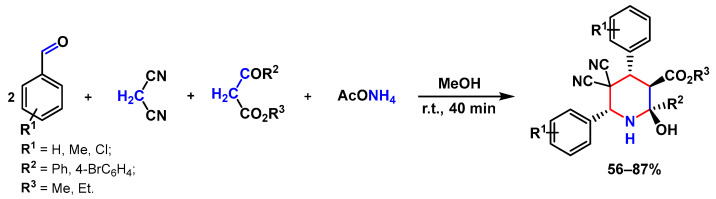
Stereoselective one-pot pseudo five-component synthesis of poly-substituted piperidinols.

The scope of the described reaction includes the synthesis of piperidinols (Scheme 61) [162,163]. The use of esters of 3-oxocarboxylic acid instead of aldehyde resulted in a product containing four stereocenters. Moreover, the resulting piperidinol undergoes dehydration in an acidic environment to form 1,4,5,6-tetrahydropyridine. The process passes through a previously unknown isomer of 3,4,5,6-tetrahydropyridine. You can read more about the mechanism and kinetics of the reaction here [162].

**Scheme 62:**
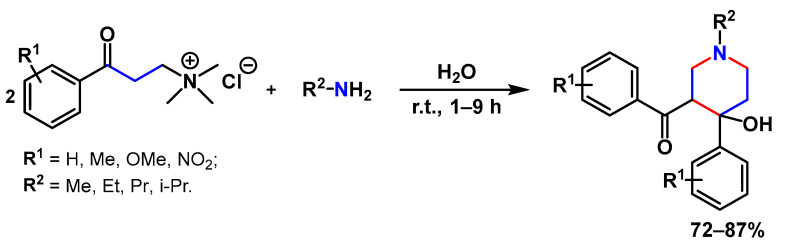
Water-mediated pseudo three-component synthesis of piperidinols.

Rashinkar and colleagues developed a new green approach to the synthesis of piperidinols [177]. The authors used previously discovered unexpected cyclization in an amine exchange reaction between primary amines and Mannich bases. Thus, they obtained a broad range of substituted piperidinols by means of water-mediated intramolecular cyclization that occurs after *bis*-aza Michael addition (Scheme 62). The products were afforded in slightly better yields when the Mannich base contained electron-withdrawing substituents. In addition, the resulting compounds showed modest anthelmintic activity.

**Scheme 63:**
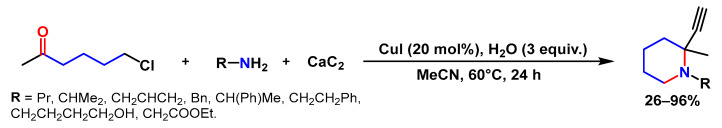
Three-component synthesis of piperidines with the acetylene group.

An interesting approach to piperidines was proposed by Tehrani et al. (Scheme 63) [178]. The authors used calcium carbide as an acetylene source and drying agent promoting the formation of cyclic ketimine from chlorinated ketone and amine. The researchers also proposed that the formed acetylene could react with a catalyst—cuprum(I) iodide. The addition of the resulting cuprum carbide to ketimine and protonation leads to product formation and catalyst regeneration. Piperidine with terminal alkyne can further be used in different cross-coupling reactions.

**Scheme 64:**
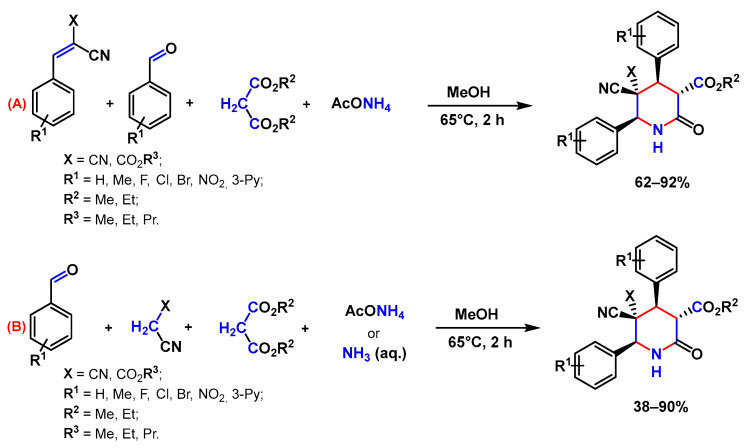
Stereoselective one-pot pseudo four-component synthesis of poly-substituted piperidinons via the Michael/Mannich (**A**) and Knoevenagel/Michael/Mannich cascade (**B**).

Vereshchagin et al. also developed a series of poly-substituted piperidinons by means of pseudo four-component synthesis (Scheme 64) [179,180]. All of the studied MCRs lead to one diastereomer with modest to high yields. Piperidinons, like piperidines, can be synthesized by the Michael/Mannich cascade (Scheme 64A) and Knoevenagel/Michael/Mannich cascade (Scheme 64B). Thus, the used approach proved to be useful for a broad variety of piperidine scaffolds.

**Scheme 65:**
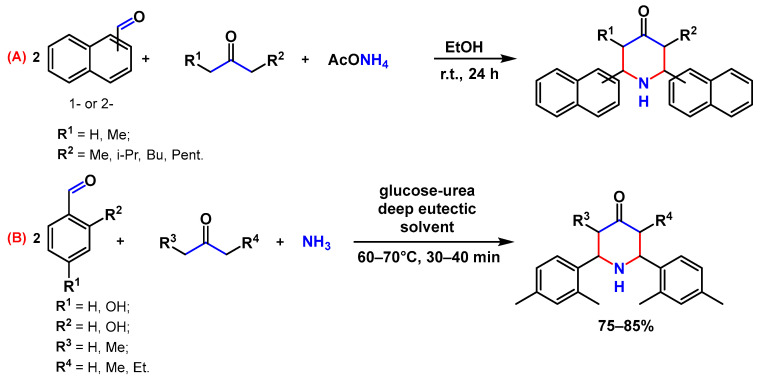
Pseudo four-component synthesis of poly-substituted piperidinons with naphthalene (**A**) and benzene (**B**) substituents.

Savithiri et al. used a similar MCR with ammonium acetate to synthesize naphthyl-substituted piperidons (Scheme 65A) [181]. The picrates derived from the obtained piperidons possessed relatively good antibacterial and antiviral properties. Ilangeswaran and colleagues designed a greener MCR for acquiring piperidinons using a glucose-urea deep eutectic solvent (Scheme 65B) [182].

**Scheme 66:**
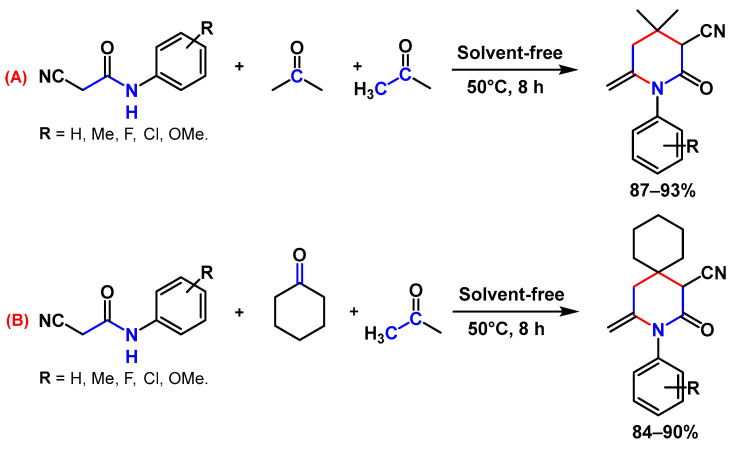
Regioselective solvent-free multicomponent synthesis of piperidinons (**A**) and spiropiperidinones (**B**).

Lin and Yan et al. represented a view on the solvent-free piperidine-promoted synthesis of piperidons from 2-cyanoacetamides and ketones (Scheme 66) [183]. The scope of the reaction covers piperidons (Scheme 66A) as well as spiropiperidinones (Scheme 66B) derivatives. It is worth noting that the reaction leads to 2-oxo-1,2,3,4-tetrahydropyridine if the amide group in 2-cyanoacetamides possesses either an alkyl or hydrogen substituent instead of aryl. Therefore, the described MCR is regioselective.

## 3. Pharmacological Applications of Piperidine Derivatives

The piperidine cycle is utterly common in pharmaceuticals. Its derivatives are used in over twenty drug classes [184], including anticancer agents [18,185,186,187,188,189], drugs for Alzheimer’s disease therapy [46], antibiotics [190], analgesics [17,191], antipsychotics [19,192,193], antioxidants [15,194], etc. (Figure 3).

**Figure 4:**
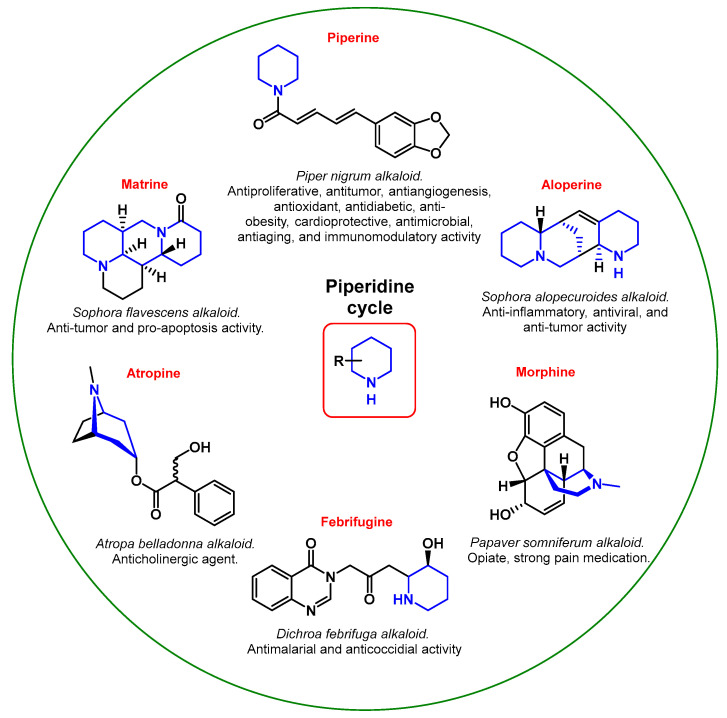
Pharmacological properties of natural piperidine derivatives.

Moreover, piperidines are also a part of many alkaloids showing biological activity (Figure 4). For example, the well-known atropine (used clinically for the treatment of vomiting, nausea, and bradycardia [195]; an effective agent for slowing the development of myopia [196]) and morphine (analgesic for severe pain relief [197]; used as a third-line therapy in the treatment of neuropathic pain [198]) contain a fused piperidine ring.

Piperine, a derivative of piperidine and the main active chemical component of black pepper, is attracting more and more attention from researchers, despite the fact that it was discovered more than 200 years ago. It is believed that piperine has a broad scope of beneficial biological properties, from antibacterial to anticancer [199,200,201,202,203]. Aloperine and Matrine—an alkaloid of the Sophora containing two fused piperidine rings at once—and their derivatives showed antiviral, anti-inflammatory, and antitumor properties [204,205]. Febrifugine and its synthetic analog halofuginone are efficiently used as antiparasitic drugs [20].

Along with already known drugs, the scientific community constantly proposes new biologically active piperidine scaffolds. Further, we will discuss recent discoveries in the biological evaluation of synthetic potential drugs containing the piperidine moiety. Particular attention was paid to four pharmaceutical groups: cancer (pro-tumorigenic receptor inhibitors, apoptosis initiators), infectious and parasitic diseases (biocides), Alzheimer’s disease (anticholinergics), and neuropathic pain (analgesics). The choice of drug groups was based on current trends and relevance in the medical community.

### 3.1. Cancer Therapy

Cancer is one of the biggest health problems worldwide, with nearly 10 million deaths reported in 2020 according to WHO. A lot of resources are spent on the development of new drugs for fighting cancer, but despite all efforts, innate and acquired resistance mechanisms are often observed [206]. Therefore, screening for new developments and breakthroughs in this area is very important and relevant.

Piperidine moieties are often used in anticancer drug construction [185,189]. Herein, the recent proposals and developments of scientists on this subject will be briefly discussed.

Arumugam et al. synthesized spirooxindolopyrrolidine-embedded piperidinone **1** with potential anticancer activity through three-component 1,3-dipolar cycloaddition and subsequent enamine reaction [207]. The resulting product showed slightly better cytotoxicity and apoptosis induction in the FaDu hypopharyngeal tumor cells model than the reference drug bleomycin. The authors followed the “escape from flatland” approach, which was popularized throughout recent years [208,209,210] and was successfully used in the development of anti-cancer agents [211,212,213]. This approach suggests that more saturated and three-dimensional structures will interact better with binding sites of proteins. Therefore, the authors reasoned that the spirocyclic structure played a key role in the biological activity of compound **1**.

Li et al. developed IκB kinase (IKKb) inhibitor **2** as an EF24 analog [214]. EF24 is a piperidinone derivative with potential activity against lung, breast, ovarian, and cervical cancer [215,216]. The activation of IKKb is one of the major factors of NF-κB transcription, which induces chronic inflammation in carcinomas, leading to desmoplasia and neoplastic progression [217]. The new analog **2** possessed better IKKb inhibitory properties than the reference drug. The active component with the piperidine moiety developed a stable hydrophobic interaction with the IKKb catalytic pocket. The structure–activity relationship shows that the presence of a nitrogen atom in the cycle is optimal, and any substitution in the ketone bridge is not favorable for IKKb inhibition.

A series of 2-amino-4-(1-piperidine) pyridine derivatives **3,** as the clinically Crizotinib-resistant anaplastic lymphoma kinase (ALK) and c-ros oncogene 1 kinase (ROS1) dual inhibitor, was designed by Zhang et al. [218]. ALK was originally discovered in anaplastic large cell lymphoma as a transmembrane receptor tyrosine kinase [219]. It was found that ALK is involved in the development of non-small cell lung cancer, neuroblastoma, diffuse large B-cell lymphoma, anaplastic thyroid cancer, rhabdomyosarcoma, ovarian cancer, esophageal squamous cell, colorectal, and breast carcinomas, etc. [220,221]. ROS1 was discovered more recently as a similar enzyme to ALK. ROS1 rearrangements were identified in glioblastoma, cholangiocarcinoma, gastric cancer, ovarian cancer, soft-tissue sarcomas, breast cancer etc. [222]. Crizotinib—the first approved ALK/ROS1 dual inhibitor—also includes the piperidine moiety [223]. Despite the fact that the piperidine fragment did not form any bonds with the active site of the receptor, its introduction was the most optimal for the desired pharmacological properties compared to other substituents. In the case of substance 3, piperidine derivatives (namely, (S or R)-ethyl piperidine-3-carboxylate) were used as the starting components for chiral optimization [218]. The piperidine ring was essential for chiral optimization. Piwnica-Worms et al. obtained radiolabeled fluoro-analogs of the commercial ALK inhibitors crizotinib **4**, alectinib **5**, and ceritinib **6** [224]. The products have a potential for brain metastases treatment due to their enhanced CNS pharmacokinetic properties. It is worth noting that the introduction of fluoroethyl groups did not affect the inhibitory properties of parent drugs, while it enhanced their ability to pass through blood–brain barrier.

Onnis et al. conducted a synthesis of benzenesulfonamide with a piperidinyl-hydrazidoureido linker as potent carbonic anhydrase (CA) II (**7**), IX (**8**), and XII (**9**) inhibitors [225]. CAs are metalloenzymes localized in the cytosol, mitochondria, membranes, and secreted substances of living organisms. CAs are involved in the catalysis of chemical processes (the hydration of carbon dioxide to bicarbonate, the conversion of cyanate to carbamic acid, etc.) and esterase activity [226]. Two out of the sixteen known types of CA (CA IX and CA XII) are found in vertebrate tumor cells. Their inhibition is an effective way to control the growth, progression, and metastasis of cancerous tumors [227]. Currently, the leading compound among CA IX and XII inhibitors is SLC-0111, which is in phase I/II of clinical trials for the management of hypoxic tumors [228,229]. The authors used SLC-0111 as the parent drug, incorporating a piperidinyl-hydrazidoureido linker in its structure to improve binding selectivity with CA. The piperidine residue was also introduced to assess rigidity [225].

Benzoylpiperidine scaffold **10** with antitumor activity via monoacylglycerol lipase (MAGL) inhibition was constructed by Granchi et al. [230,231]. Fluorine atoms and the meta-substitution of the benzene ring enhanced the inhibition properties. MAGL is responsible for the inactivation of the brain’s endocannabinoid 2-arachidonoylglycerol. Moreover, MAGL indirectly controls the levels of free fatty acids, as well as other lipids with pro-inflammatory or pro-oncogenic effects, therefore causing pain and cancer progression [232]. Sekhar et al. developed spirochromanone analog **11** with significant activity against the breast cancer cell line and Murine melanoma, as well as the ability to induce apoptosis [233]. The authors combined known pharmacophore structures to achieve the best anti-proliferative and anti-cancer effects.

**Figure 5:**
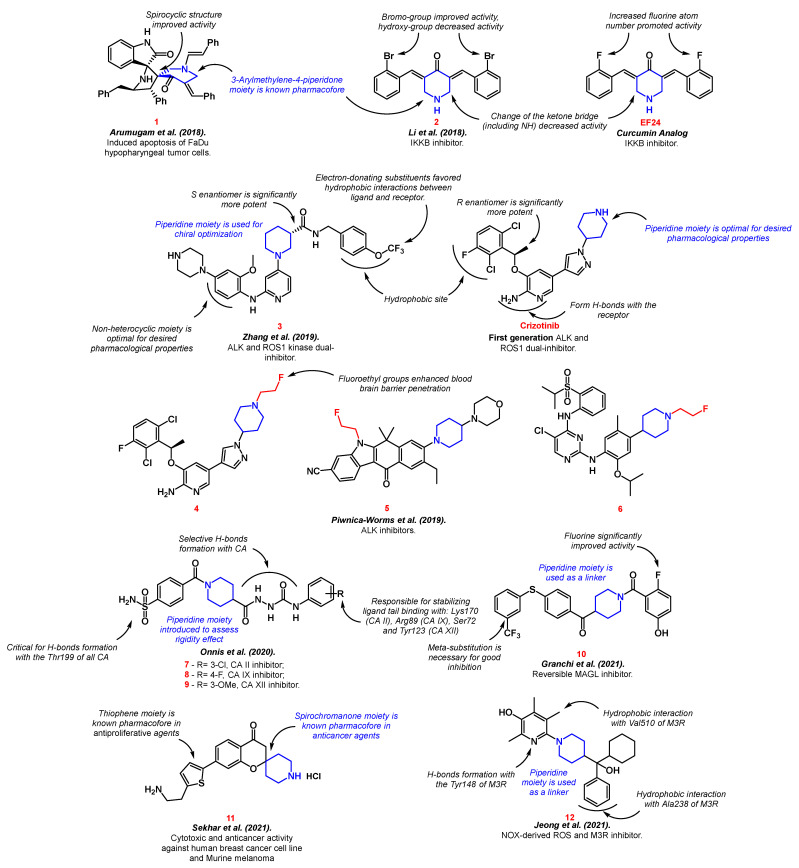
Structure–activity relationship of piperidine derivatives with anticancer activity [207,214,218,223,224,225,230,231,233,234].

Jeong et al. synthesized piperidine-embedded anticancer agents with particularly good activity on androgen-refractory cancer cell lines (ARPC) [234]. The authors showed that compound **12** was a ligand to the M3 muscarinic acetylcholine receptor (M3R), which is presented in ARPC (Figure 5). M3R activation stimulates cell proliferation, resistance to apoptosis, and metastasis and is responsible for the early progression and invasion of colorectal cancer tumors [235,236,237].

### 3.2. Alzheimer Disease Therapy

Alzheimer disease is one of the most lethal and burdening illnesses of the last century. It has no definite treatment other than symptomatic treatment and results in death 6 years after diagnosis, on average [238]. The oldest theory of Alzheimer’s disease is the cholinergic hypothesis, which suggests that the illness is caused by the loss of cholinergic innervation [239].

The neurotransmitter acetylcholine is one of many vital components for normal brain function. Deficiency of the cholinergic system has been observed in the brains of Alzheimer’s disease patients, leading to the pathophysiology of learning and memory impairment [240]. The main goal of modern therapy is to maintain the level of acetylcholine through the inhibition of cholinesterases: acetylcholinesterase (ACHe) and butyrylcholinesterase (BuCHe) [241]. Currently, the leading drug among acetylcholinesterase inhibitors is Donepezil, a piperidine derivative.

Martinez et al. proposed indolylpiperidine analog **13** of Donepezil [242]. The active agent was capable of inhibiting both acetylcholinesterase (AChE) and butyrylcholinesterase (BuChE) enzymes. Moreover, the authors discovered unusual conformational changes in the molecule depending on the binding site. Thus, compound **13** was extended in AChE interaction and flopped in BuChE interaction. Liu et al. expanded this field with 4-N-phenylaminoquinoline derivative 14 [243] via piperidine moiety introduction to a previously reported lead compound [244]. Piperidine incorporation improved the brain exposure of the resulting dual inhibitor. In addition, the compound showed antioxidant and metal chelating properties.

In 2018, Gobec et al. designed selective BuChe inhibitor **15** [245]. Two cationic nitrogen atoms were essential for selectivity and good inhibition properties. Further, the authors conducted a detailed structure–activity relationship study of *N-*alkylpiperidine carbamates [246]. Structures with an *N-*benzyl moiety were superior in cholinesterase inhibition, and a terminal alkyne group was essential for efficient monoamine oxidase B inhibition. Thus, compounds **16–18** were selected as the best in the series. Moreover, a study by Malawska and Gobec outlined a multi-targeted approach to Alzheimer’s disease treatment. Novel 1-Benzylpyrrolidine-3-amine derivatives with piperidine groups **19** and **20** expressed both antiaggregatory and antioxidant effects [247]. Along with dual cholinesterase inhibition, compounds **19–20** also targeted the beta secretase enzyme.

Beta secretase is also known as beta-site amyloid precursor protein cleaving enzyme-1 (BACE-1). It has been established that the inhibition of BACE-1 prevents the accumulation of amyloid beta [248,249]. According to the current concept of Alzheimer’s disease based on the amyloid hypothesis, deposits of amyloid beta and tau proteins cause neurodegeneration and cognitive impairment [250].

**Figure 6:**
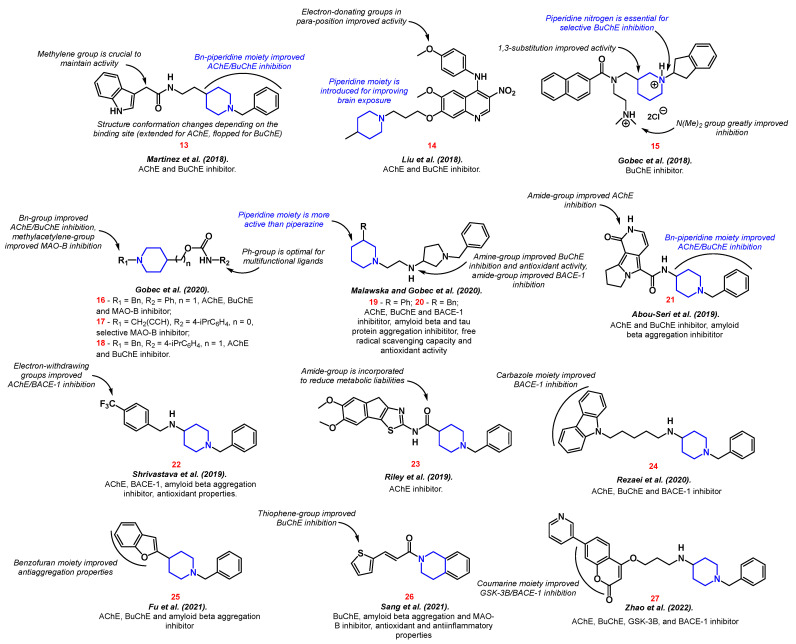
Piperidine derivatives as potential drugs for Alzheimer disease therapy [242,243,244,245,246,247,252,253,254,255,256,257,258].

The benzyl-piperidine group (Donepezil-like) is often a necessary part for the successful inhibition of cholinesterase receptors. The AChE enzyme includes two active anionic binding sites: catalytic and peripheral. The benzyl-piperidine group provides good binding to the catalytic site, interacting with Trp84, Trp279, Phe330, and Phe331 [251]. Therefore, the selection of various substituents on top of the benzyl-piperidine residue is a well-established approach in the synthesis of new active agents for combatting Alzheimer’s disease. Thus, pyrrolizine 21 [252], fluorine 22 [253], thiazole 23 [254], indoline 24 [255], benzofuran 25 [256], thiophene 26 [257], and chromene 27 [258] groups have been effectively incorporated and biologically evaluated by various authors. The structure–activity relationship is shown in Figure 6.

It is worth noting that research on multifunctional active agents is prevalent compared to compounds that affect only one target. Therefore, along with inhibitors of cholinesterase receptors, attention is also paid to inhibitors of monoamine oxidase **16–17**, **26** [246,257], amyloid beta and tau protein aggregation **19–20**, **22**, **25–26** [247,253,256,257], BACE-1 **19–20**, **22**, **24**, **27** [247,253,255,258], as well as the presence of anti-inflammatory 26 [257], anti-radical **19–20** [247], and antioxidant properties **19–20**, **22**, **26** [247,253,257]. 

Drawing conclusions from the analyzed data, it can be said that the piperidine group affects the inhibition of cholinesterase receptors and serves as a constructing (linker) part.

### 3.3. Biocides

Biocides are chemical compounds designed to neutralize, suppress, or prevent the action of harmful organisms, namely, pathogenic bacteria, fungi, viruses, parasites, etc. [259]. As noted earlier, piperidine derivatives find use in this class of pharmaceuticals. 

In recent years, a number of works on the topic can be noted. However, due to the wide variety of human pathogens, it is not possible to point out one template structure for all types of activities.

Thus, piperidine moieties were represented in structures with antifungal properties **28–30**. Compounds containing tartaric acid fragment **28–29** inhibited chitin synthase, therefore suppressing a growth of five fungi strains (*C. albicans* ATCC 76615, *A. fumigatus* GIMCC 3.19, *C. albicans* ATCC 90023, *C. neofonmans* ATCC 32719, *A. flavus* ATCC 16870) [260]. The resulting compounds combined two pharmacophores: 2,8-Diazaspiro [4.5]decane-1-one and a tartaric acid residue with a substituted aminobenzene. When designing the structure, the authors were guided by the “escape from flatness” theory (which was mentioned earlier) and the enzyme inhibition potential of the chosen moieties. Long-tailed 4-aminopiperidines **30** have proven to be effective against fungi of the genus Aspergillus and Candida via fungal ergosterol biosynthesis inhibition [261]. Ergosterol is one of the most abundant fungal cell membrane sterols. It is responsible for membrane permeability and fluidity [262].

Piperidine-containing fluoroquinolones analogs, namely, bafloxacins **31**, were proposed by Liu et al. [263]. Along with the good inhibition values on MRSA, *P. aeruginosa*, and *E. coli*, the new compounds showed good biocompatibility and potential two-targeted action via cell walls destruction and interaction with IV-DNA and DNA gyrase. Piperidinyl “tails” structures **32** possessed inhibition properties against streptomycin-starved Mycobacterium tuberculosis 18b (SS18b) and H37Rv strains [264]. Compound **32** consists of various known tubercular pharmacofores with piperidine as a linker.

The benzyl-piperidines activity against different viruses was shown. Thus, 4,4-disubstituted *N-*benzyl piperidines **33** inhibited the H1N1 influenza virus through specific hemagglutinin fusion peptide interaction [265]. Nayagam et al. discovered the potential inhibitor of SARS-CoV2 with piperidine core **34** [266]. Compound **34** possessed a better binding affinity with the SARS-CoV2 main protease than Remdesivir, with five binding pockets interaction compared to two.

**Figure 7:**
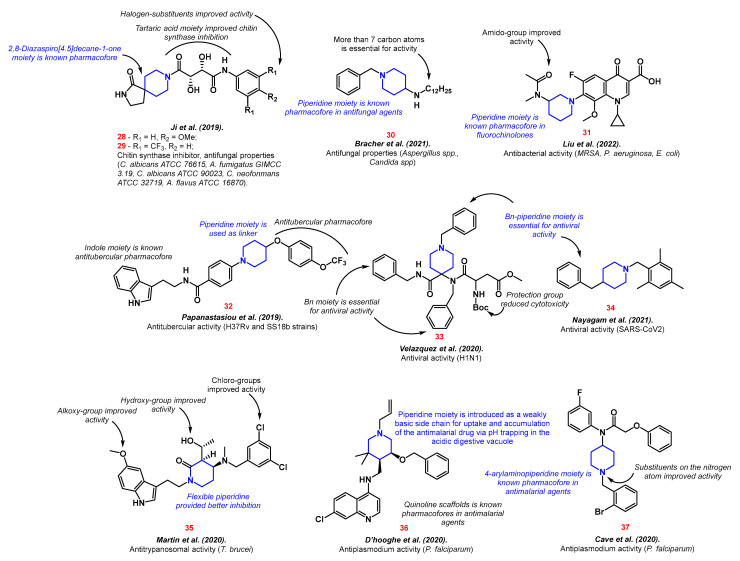
Piperidine derivatives with antimicrobial activity [260,261,263,264,265,266,267,268,269].

Compounds with a piperidine backbone structure have shown antiparasitic properties on *T. brucei* (the main cause of African trypanosomiasis) **35** [267] and *P. falciparum* (the cause of the deadliest type of malaria) **36–37** [268,269] (Figure 7).

### 3.4. Neuropathic Pain Therapy

Neuropathic pain occurs as a result of the pathological excitation of neurons in the peripheral or central nervous system, which is caused by neurological diseases with damage to peripheral fibers and central neurons [270]. The modern approach to the treatment of neuropathic pain includes three lines of pharmacotherapy. Most of the piperidine derivatives are part of opioids, which are the second and, in some cases, the third line of treatment [198].

Opioid receptors are divided into four similar types: μ-opioid (MOR), δ-opioid (DOR), κ-opioid (KOR), and nociceptin/orphanin FQ peptide receptor (NOP) [271,272]. MOR and DOR are the main targets of opioid agonists. MOR agonists cause euphoria and help with coping with stress; however, their use causes serious side effects and physical dependence, leading to overdose [273]. One of the main synthetic piperidine-containing opioids is fentanyl.

Most often, opioid derivatives serve as the starting point for the discovery of new types of analgesics. Thus, derivatives of norsufentanil with amino acids **38** were developed [274], the synthesis of the main metabolites of carfentanil **39** was reproduced [275], and new analogs of tramadol **40** were proposed [276]. All compounds showed a strong affinity for MOR.

In order to achieve a dual effect ligand, Lee et al. created a hybrid based on Pethidine, also known as meperidine, and a transient receptor potential cation channel subfamily V member 1 (TrpV1) antagonist **41** [277]. TrpV1 functions are widely linked to the generation of pain [278]. This combination potentially increases the anti-inflammatory effect and treatment efficiency.

Navarrete-Vázquez et al. developed a haloperidol analog **42** as a σ1 receptor antagonist [279]. The σ1 receptor plays a role in various regulatory processes, including pain reduction [280]. Therefore, Chen et al. proposed novel piperidine propionamide derivatives **43–44** as dual agonists of μ-opioid and σ1 receptors [281,282].

**Figure 8:**
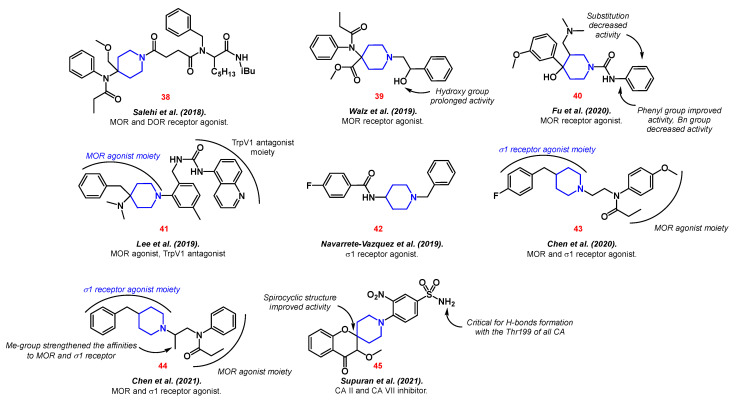
Piperidine derivatives as potential drugs for neuropathic pain disorders [274,275,276,277,279,281,282,283].

Lastly, CA inhibition is another prominent therapeutic target in neuropathic pain treatment. Thus, Supuran et al. synthetized piperidine-embedded 4-oxo-spirochromanes **45** with high activity against CA II and CA VII (Figure 8) [283].

## 4. Conclusions

Piperidines play an important role in both chemistry and medicine. In this review, attention has been paid to both of these applications of piperidines.

We can safely say that approaches to the piperidine synthesis have improved significantly in recent years. There is a shift towards shortening the synthesis steps number. Thus, methodologies are used for the simultaneous functionalization/hydrogenation of pyridines, the functionalization/cyclization of unsaturated amines, as well as multicomponent cascade reactions with the formation of several new C-N and C-C bonds at once. Scientists are successfully applying new routes for the stereoselective synthesis of piperidines, using catalysis with transition metal cores such as palladium, rhodium, ruthenium, nickel, chromium, platinum, cobalt, etc. complexes, as well as organocatalysts (amino-hydroquinine, chiral NHC-catalyst, boron complexes). Moreover, new green reactions have also been proposed: non-toxic iron catalysis, water-initiated processes, environmentally benign electrolytic methods, solvent-free reactions, etc.

In the medical part, examples of the pharmacological use of synthetic piperidines and natural piperidine derivatives in the composition of alkaloids are given. We have shed light on some of the latest scientific achievements in the synthesis of biologically active agents based on piperidines against socially significant diseases. Overall, piperidines can act both as a part of the pharmacophore, interacting directly with the active site of enzymes, and as a convenient building block to achieve the desired conformation and physical properties of pharmaceuticals.

The current review was designed to highlight the wide range of synthesis and medical applications of piperidines, as one of the most important groups of chemicals.

## Data Availability

Not applicable.

## Abbreviations

Substituents	
Ac	Acetyl
Ar	Aromatics
Bn	Benzyl
Boc	tert-Butyloxycarbonyl
Bu	Butyl
Cbz	Benzyloxycarbonyl
COD	Cyclooctadiene
coe	Cyclooctene
Cp	Cyclopentadienyl
dba	Dibenzylideneacetone
Et	Ethyl
Hbpin	Pinacolborane
hex	Hexyl
IMes	1,3-bis(2,4,6-trimethylphenyl)imidazol-2-ylidene
L	Ligand
Me	Methyl
Ms	Methanesulfonyl
Napht	Naphtyl
nbd	Norbornadiene
Ns	Nosyl
Ph	Phenyl
Phen	Phenanthroline
Pent	Pentyl
Piv	Pivaloyl
PMP	p-Methoxyphenyl
ppy	Phenylpyridine
Pr	Propyl
PS	Polystyrene
Py	Pyridine
Tf	Triflate
TPP	Tetraphenylporphine
Ts	Toluenesulfonyl
Reagents/Solvents	
DBAD	Dibenzyl azodicarboxylate
DBU	1,8-Diazabicyclo [5.4.0]undec-7-ene
DIBAL-H	Diisobutylaluminium hydride
DCE	Dichloroethane
DCM	Dichloromethane
DMA	Dimethylacetamide
DMF	Dimethylformamide
DMPU	N,N′-Dimethylpropyleneurea
DMSO	Dimethyl sulfoxide
HATU	2-(7-aza-1H-benzotriazole-1-yl)-1,1,3,3-tetramethyluronium hexafluorophosphate
HFIP	Hexafluoroisopropanol
KHDMS	Potassium bis(trimethylsilyl)amide
LiHDMS	Lithium bis(trimethylsilyl)amide
PEG	Polyethylene glycol
PIDA	Phenyliodine(III) diacetate
STAB	Sodium triacetoxyborohydride
TBAF	Tetra-n-butylammonium fluoride
TBHP	tert-Butyl hydroperoxide
TFA	Trifluoroacetic acid
TFE	Trifluoroethanol
THF	Tetrahydrofuran
TIPS	Triisopropyl silane
TMEDA	Tetramethylethylenediamine
TMS	Tetramethylsilane
Term:	
dr	Diastereomeric Ratio
ee	Enantiomeric Excess
er	Enantiomeric Ratio
HAT	Hydrogen Atom Transfer
IMAMR	Intramolecular Aza-Michael Reactions
MCR	Multicomponent Reaction
MOC	Memory of Chirality
Medical:	
11β-HSD1	11β-Hydroxysteroid Dehydrogenase Type 1
Ache	Acetylcholinesterase
ALK	Anaplastic Lymphoma Kinase
ARPC	Androgen-Refractory Cancer Cell Lines
BACE-1	Beta-secretase 1
Buche	Butyrylcholinesterase
CA	Carbonic Anhydrase
CGRP	Calcitonin Gene-Related Peptide Receptor
CNS	Central Nervous System
DNA	Deoxyribonucleic Acid
Ikkb	Iκb Kinase
M3R	M3 Muscarinic Acetylcholine Receptor
MAGL	Monoacylglycerol Lipase
MAO-B	Monoamine Oxidase B
MRSA	Methicillin-Resistant Staphylococcus Aureus
NF-κB	Nuclear factor kappa-light-chain-enhancer of activated B cells
ROS1	C-Ros Oncogene 1
Trpv1	Transient Receptor Potential Cation Channel Subfamily V Member 1
WHO	World Health Organization

## References

[1] Sandmeier T., Carreira E.M. (2020). Modern Catalytic Enantioselective Approaches to Piperidines. Synthetic Approaches to Nonaromatic Nitrogen Heterocycles.

[2] Díez-Poza C., Barbero H., Diez-Varga A., Barbero A., Gribble G.W., Joule J.A. (2018). Chapter 2–The Silyl-Prins Reaction as an Emerging Method for the Synthesis of Heterocycles. Progress in Heterocyclic Chemistry.

[3] Kaur G., Devi M., Kumari A., Devi R., Banerjee B. (2018). One-Pot Pseudo Five Component Synthesis of Biologically Relevant 1,2,6-Triaryl-4-arylamino-piperidine-3-ene-3-carboxylates: A Decade Update. ChemistrySelect.

[4] Kaur G., Devi P., Thakur S., Kumar A., Chandel R., Banerjee B. (2019). Magnetically Separable Transition Metal Ferrites: Versatile Heterogeneous Nano-Catalysts for the Synthesis of Diverse Bioactive Heterocycles. ChemistrySelect.

[5] Park S. (2019). B(C6F5)3-Catalyzed sp3 C—Si Bond Forming Consecutive Reactions. Chin. J. Chem..

[6] Sbei N., Listratova A.V., Titov A.A., Voskressensky L.G. (2019). Recent Advances in Electrochemistry for the Synthesis of N-Heterocycles. Synthesis.

[7] Vargas D.F., Larghi E.L., Kaufman T.S. (2019). The 6π-azaelectrocyclization of azatrienes. Synthetic applications in natural products, bioactive heterocycles, and related fields. Nat. Prod. Rep..

[8] Bates R.W., Ko W., Barát V. (2020). The endo-aza-Michael addition in the synthesis of piperidines and pyrrolidines. Org. Biomol. Chem..

[9] Kaur N., Ahlawat N., Verma Y., Grewal P., Bhardwaj P., Jangid N.K. (2020). Cu-assisted C–N bond formations in six-membered N-heterocycle synthesis. Synth. Commun..

[10] Neto J.S.S., Zeni G. (2020). Ten years of progress in the synthesis of six-membered N-heterocycles from alkynes and nitrogen sources. Tetrahedron.

[11] Yu J., Luan X. (2021). Hydroxylamines as One-Atom Nitrogen Sources for Metal-Catalyzed Cycloadditions. Synthesis.

[12] Maurya R.K., Sharma D., Kumari S., Chatterjee R., Khatravath M., Dandela R. (2022). Recent Advances in Transition Metal-Catalyzed Domino-Cyclization Strategies for Functionalized Heterocyclic/Carbocyclic Compounds. ChemistrySelect.

[13] Slivka M., Korol N. (2022). Synthesis of mononuclear heterocycles via electrophilic cyclization. Mon. Für Chem.-Chem. Mon..

[14] Liu G.-Q., Opatz T., Scriven E.F.V., Ramsden C.A. (2018). Chapter Two-Recent Advances in the Synthesis of Piperidines: Functionalization of Preexisting Ring Systems. Advances in Heterocyclic Chemistry.

[15] Mezeiova E., Spilovska K., Nepovimova E., Gorecki L., Soukup O., Dolezal R., Malinak D., Janockova J., Jun D., Kuca K. (2018). Profiling donepezil template into multipotent hybrids with antioxidant properties. J. Enzym. Inhib. Med. Chem..

[16] Waters S., Tedroff J., Ponten H., Klamer D., Sonesson C., Waters N. (2018). Pridopidine: Overview of Pharmacology and Rationale for its Use in Huntington’s Disease. J. Huntingt. Dis..

[17] Martinelli D., Bitetto V., Tassorelli C. (2021). Lasmiditan: An additional therapeutic option for the acute treatment of migraine. Expert Rev. Neurother..

[18] McLornan D.P., Pope J.E., Gotlib J., Harrison C.N. (2021). Current and future status of JAK inhibitors. Lancet.

[19] Rathore A., Asati V., Kashaw K.S., Agarwal S., Parwani D., Bhattacharya S., Mallick C. (2021). The Recent Development of Piperazine and Piperidine Derivatives as Antipsychotic Agents. Mini-Rev. Med. Chem..

[20] Gill J., Sharma A. (2022). Prospects of halofuginone as an antiprotozoal drug scaffold. Drug Discov. Today.

[21] Wozniak B., Tin S., de Vries J.G. (2019). Bio-based building blocks from 5-hydroxymethylfurfural via 1-hydroxyhexane-2,5-dione as intermediate. Chem. Sci..

[22] Wang Y., Zhao D., Rodríguez-Padrón D., Len C. (2019). Recent Advances in Catalytic Hydrogenation of Furfural. Catalysts.

[23] Xu C., Paone E., Rodríguez-Padrón D., Luque R., Mauriello F. (2020). Recent catalytic routes for the preparation and the upgrading of biomass derived furfural and 5-hydroxymethylfurfural. Chem. Soc. Rev..

[24] Journot G., Neier R., Gualandi A. (2021). Hydrogenation of Calix[4]pyrrole: From the Formation to the Synthesis of Calix[4]pyrrolidine. Eur. J. Org. Chem..

[25] Hua T.-B., Xiao C., Yang Q.-Q., Chen J.-R. (2020). Recent advances in asymmetric synthesis of 2-substituted indoline derivatives. Chin. Chem. Lett..

[26] Yan Q., Xiao G., Wang Y., Zi G., Zhang Z., Hou G. (2019). Highly Efficient Enantioselective Synthesis of Chiral Sulfones by Rh-Catalyzed Asymmetric Hydrogenation. J. Am. Chem. Soc..

[27] Schlepphorst C., Wiesenfeldt M.P., Glorius F. (2018). Enantioselective Hydrogenation of Imidazo[1,2-a]pyridines. Chem.–A Eur. J..

[28] Li W., Wollenburg M., Glorius F. (2018). Enantioselective synthesis of 2-oxazolidinones by ruthenium(ii)–NHC-catalysed asymmetric hydrogenation of 2-oxazolones. Chem. Sci..

[29] Liu W., Sahoo B., Junge K., Beller M. (2018). Cobalt Complexes as an Emerging Class of Catalysts for Homogeneous Hydrogenations. Acc. Chem. Res..

[30] Gunasekar R., Goodyear R.L., Proietti Silvestri I., Xiao J. (2022). Recent developments in enantio- and diastereoselective hydrogenation of N-heteroaromatic compounds. Org. Biomol. Chem..

[31] Chen F., Li W., Sahoo B., Kreyenschulte C., Agostini G., Lund H., Junge K., Beller M. (2018). Hydrogenation of Pyridines Using a Nitrogen-Modified Titania-Supported Cobalt Catalyst. Angew. Chem. Int. Ed..

[32] Bourriquen F., Hervochon J., Qu R., Bartling S., Rockstroh N., Junge K., Fischmeister C., Beller M. (2022). Diastereoselective hydrogenation of arenes and pyridines using supported ruthenium nanoparticles under mild conditions. Chem. Commun..

[33] Mao S., Ryabchuk P., Dastgir S., Anwar M., Junge K., Beller M. (2022). Silicon-Enriched Nickel Nanoparticles for Hydrogenation of N-Heterocycles in Aqueous Media. ACS Appl. Nano Mater..

[34] Meltzer H.Y., Sumiyoshi T., Jayathilake K. (2001). Melperone in the treatment of neuroleptic-resistant schizophrenia. Psychiatry Res..

[35] Qu B., Mangunuru H.P.R., Tcyrulnikov S., Rivalti D., Zatolochnaya O.V., Kurouski D., Radomkit S., Biswas S., Karyakarte S., Fandrick K.R. (2018). Enantioselective Synthesis of α-(Hetero)aryl Piperidines through Asymmetric Hydrogenation of Pyridinium Salts and Its Mechanistic Insights. Org. Lett..

[36] Cui C.-X., Chen H., Li S.-J., Zhang T., Qu L.-B., Lan Y. (2020). Mechanism of Ir-catalyzed hydrogenation: A theoretical view. Coord. Chem. Rev..

[37] Qu B., Wei X., Zeng X., Yang B.-S., Desrosiers J.-N., Savoie J., Wang J., Marsini M.A., Li Z., Haddad N. (2022). Large-Scale Enantioselective Reduction of 2,3-Disubstituted Indenopyridine Enables a Practical Manufacturing Process for an 11β-HSD-1 Inhibitor. Org. Process Res. Dev..

[38] Gregory S., Hill D., Grey B., Ketelbey W., Miller T., Muniz-Terrera G., Ritchie C.W. (2020). 11β-hydroxysteroid dehydrogenase type 1 inhibitor use in human disease-a systematic review and narrative synthesis. Metabolism.

[39] Nairoukh Z., Wollenburg M., Schlepphorst C., Bergander K., Glorius F. (2019). The formation of all-cis-(multi)fluorinated piperidines by a dearomatization–hydrogenation process. Nat. Chem..

[40] Wagener T., Heusler A., Nairoukh Z., Bergander K., Daniliuc C.G., Glorius F. (2020). Accessing (Multi)Fluorinated Piperidines Using Heterogeneous Hydrogenation. ACS Catal..

[41] Wagener T., Lückemeier L., Daniliuc C.G., Glorius F. (2021). Interrupted Pyridine Hydrogenation: Asymmetric Synthesis of δ-Lactams. Angew. Chem. Int. Ed..

[42] Subota A.I., Lutsenko A.O., Vashchenko B.V., Volochnyuk D.M., Levchenko V., Dmytriv Y.V., Rusanov E.B., Gorlova A.O., Ryabukhin S.V., Grygorenko O.O. (2019). Scalable and Straightforward Synthesis of All Isomeric (Cyclo)alkylpiperidines. Eur. J. Org. Chem..

[43] Subota A.I., Ryabukhin S.V., Gorlova A.O., Grygorenko O.O., Volochnyuk D.M. (2019). An approach to the synthesis of 3-substituted piperidines bearing partially fluorinated alkyl groups. J. Fluor. Chem..

[44] Pitna D.B., Tanaka N., Usuki T. (2021). Pd/C-catalyzed one-pot Suzuki-Miyaura cross-coupling/hydrogenation of pyridine derivatives. J. Heterocycl. Chem..

[45] Tanaka N., Usuki T. (2020). Can Heteroarenes/Arenes Be Hydrogenated Over Catalytic Pd/C Under Ambient Conditions?. Eur. J. Org. Chem..

[46] Li Q., He S., Chen Y., Feng F., Qu W., Sun H. (2018). Donepezil-based multi-functional cholinesterase inhibitors for treatment of Alzheimer′s disease. Eur. J. Med. Chem..

[47] Wang W.-T., Qian H., Wu J.-W., Chen X.-W., Li J.-Q. (2019). Synthesis and antidepressant-like activity of novel alkoxy-piperidine derivatives targeting SSRI/5-HT1A/5-HT7. Bioorganic Med. Chem. Lett..

[48] Yao K., Yuan Q., Qu X., Liu Y., Liu D., Zhang W. (2019). Pd-catalyzed asymmetric allylic substitution cascade using α-(pyridin-1-yl)-acetamides formed in situ as nucleophiles. Chem. Sci..

[49] Clarke J.J., Maekawa Y., Nambo M., Crudden C.M. (2021). Borenium-Catalyzed Reduction of Pyridines through the Combined Action of Hydrogen and Hydrosilane. Org. Lett..

[50] Yang Z.-Y., Luo H., Zhang M., Wang X.-C. (2021). Borane-Catalyzed Reduction of Pyridines via a Hydroboration/Hydrogenation Cascade. ACS Catal..

[51] Cao V.D., Mun S.H., Kim S.H., Kim G.U., Kim H.G., Joung S. (2020). Synthesis of Cyclic Amidines from Quinolines by a Borane-Catalyzed Dearomatization Strategy. Org. Lett..

[52] Cao V.D., Jo D.G., Kim H., Kim C., Yun S., Joung S. (2020). Utilization of Borane-Catalyzed Hydrosilylation as a Dearomatizing Tool: Six-Membered Cyclic Amidine Synthesis from Isoquinolines and Pyridines. Synthesis.

[53] Gandhamsetty N., Park S., Chang S. (2015). Selective Silylative Reduction of Pyridines Leading to Structurally Diverse Azacyclic Compounds with the Formation of sp3 C–Si Bonds. J. Am. Chem. Soc..

[54] Molinaro C., Phillips E.M., Xiang B., Milczek E., Shevlin M., Balsells J., Ceglia S., Chen J., Chen L., Chen Q. (2019). Synthesis of a CGRP Receptor Antagonist via an Asymmetric Synthesis of 3-Fluoro-4-aminopiperidine. J. Org. Chem..

[55] Qu B., Saha A., Li G., Zeng X., Wang X., Haddad N., Hou X., Lorenz J.C., Wu L., Pennino S. (2021). Large Scale Practical Synthesis of Enantiomerically Pure cis-4-Amino-3-fluoro-1-methylpiperidine via Rhodium-Catalyzed Asymmetric Hydrogenation of a Tetrasubstituted Fluoroalkene. Org. Process Res. Dev..

[56] Levashova E., Firsov A., Bakulina O., Peshkov A., Kanov E., Gainetdinov R.R., Krasavin M. (2021). Rare cis-configured 2,4-disubstituted 1-alkylpiperidines: Synthesized and tested against trace-amine-associated receptor 1 (TAAR1). Mendeleev Commun..

[57] Li C., Wan F., Chen Y., Peng H., Tang W., Yu S., McWilliams J.C., Mustakis J., Samp L., Maguire R.J. (2019). Stereoelectronic Effects in Ligand Design: Enantioselective Rhodium-Catalyzed Hydrogenation of Aliphatic Cyclic Tetrasubstituted Enamides and Concise Synthesis of (R)-Tofacitinib. Angew. Chem. Int. Ed..

[58] Baldwin J.E. (1976). Rules for ring closure. J. Chem. Soc. Chem. Commun..

[59] Gilmore K., Mohamed R.K., Alabugin I.V. (2016). The Baldwin rules: Revised and extended. WIREs Comput. Mol. Sci..

[60] Mendes J.A., Costa P.R.R., Yus M., Foubelo F., Buarque C.D. (2021). N-tert-Butanesulfinyl imines in the asymmetric synthesis of nitrogen-containing heterocycles. Beilstein J. Org. Chem..

[61] Díez-Poza C., Barbero A. (2017). Synthesis of O- and N-Heterocycles by Silyl-Prins Cyclization of Allylsilanes. Eur. J. Org. Chem..

[62] Feige P., de Haro T., Rusconi G., Merino E., Nevado C. (2018). Gold-catalyzed oxidative aminoesterification of unactivated alkenes. Mon. Für Chem.-Chem. Mon..

[63] Qi X., Chen C., Hou C., Fu L., Chen P., Liu G. (2018). Enantioselective Pd(II)-Catalyzed Intramolecular Oxidative 6-endo Aminoacetoxylation of Unactivated Alkenes. J. Am. Chem. Soc..

[64] Li X., Qi X., Hou C., Chen P., Liu G. (2020). Palladium(II)-Catalyzed Enantioselective Azidation of Unactivated Alkenes. Angew. Chem. Int. Ed..

[65] Liu X., Hou C., Peng Y., Chen P., Liu G. (2020). Ligand-Controlled Regioselective Pd-Catalyzed Diamination of Alkenes. Org. Lett..

[66] Suzuki S., Kamo T., Fukushi K., Tokunaga E., Sumii Y., Shibata N. (2018). Intramolecular Aminotrifluoromethanesulfinyloxylation of ω-Aminoalkenes by CF3SO2Na/Pd(OAc)2/PhI(OAc)2/tBuOCl/PivOH System. Synlett.

[67] Olszewska B., Jabłonska A., Zawisza A. (2018). Diastereoselective synthesis of 2-vinylpyrrolidines and 2-vinylpiperidines by the palladium-catalysed cyclization of amino-allylic carbonates containing a chiral protecting group. Arkivoc.

[68] Wang X., Li Z.-Q., Mai B.K., Gurak J.A., Xu J.E., Tran V.T., Ni H.-Q., Liu Z., Liu Z., Yang K.S. (2020). Controlling cyclization pathways in palladium(ii)-catalyzed intramolecular alkene hydro-functionalization via substrate directivity. Chem. Sci..

[69] Cox L., Zhu Y., Smith P.J., Christensen K.E., Sidera Portela M., Donohoe T.J. (2022). Alcohols as Alkylating Agents in the Cation-Induced Formation of Nitrogen Heterocycles. Angew. Chem. Int. Ed..

[70] Sánchez-Roselló M., Aceña J.L., Simón-Fuentes A., del Pozo C. (2014). A general overview of the organocatalytic intramolecular aza-Michael reaction. Chem. Soc. Rev..

[71] Sánchez-Roselló M., Escolano M., Gaviña D., del Pozo C. (2022). Two Decades of Progress in the Asymmetric Intramolecular aza-Michael Reaction. Chem. Rec..

[72] Guerola M., Escolano M., Alzuet-Piña G., Gómez-Bengoa E., Ramírez de Arellano C., Sánchez-Roselló M., del Pozo C. (2018). Synthesis of substituted piperidines by enantioselective desymmetrizing intramolecular aza-Michael reactions. Org. Biomol. Chem..

[73] Escolano M., Guerola M., Torres J., Gaviña D., Alzuet-Piña G., Sánchez-Rosello M., del Pozo C. (2020). Organocatalytic enantioselective synthesis of 2,5,5-trisubstituted piperidines bearing a quaternary stereocenter. Vinyl sulfonamide as a new amine protecting group. Chem. Commun..

[74] Chowdhury S., Mao J., Martynow J., Zhao H., Duffy E., Wu Y., Thakur V., Sirasani G., Tang Y., Collin F. (2019). Diastereoselective scalable synthesis of 2,6-trans-Piperidines using an aza-Michael reaction. Tetrahedron Lett..

[75] Zhang Z.-F., Gao Z.-H., Zhang C.-L., Ye S. (2021). N-heterocyclic carbene-catalyzed intramolecular aza-Michael addition of alkyl amines to α,β-unsaturated carboxylic acid: Synthesis of pyrrolidines and piperidines. Tetrahedron.

[76] Bell J.D., Harkiss A.H., Wellaway C.R., Sutherland A. (2018). Stereoselective synthesis of 2,6-trans-4-oxopiperidines using an acid-mediated 6-endo-trig cyclisation. Org. Biomol. Chem..

[77] Yamazaki S., Naito T., Tatsumi T., Kakiuchi K. (2018). Synthesis of Piperidines via Intramolecular Hydride Transfer from α-Amino sp3 Carbon Atoms to Ethenetricarboxylate-Derived Fragments and Further Cyclization. ChemistrySelect.

[78] Ma X., Hazelden I.R., Langer T., Munday R.H., Bower J.F. (2019). Enantioselective Aza-Heck Cyclizations of N-(Tosyloxy)carbamates: Synthesis of Pyrrolidines and Piperidines. J. Am. Chem. Soc..

[79] Sadanandan S., Gupta D.K. (2020). Changing stereoselectivity and regioselectivity in copper(i)-catalyzed 5-exo cyclization by chelation and rigidity in aminoalkyl radicals: Synthesis towards diverse bioactive N-heterocycles. New J. Chem..

[80] Meng Z., Zhang X., Shi M. (2021). Visible-light mediated cascade cyclization of ene-vinylidenecyclopropanes: Access to fluorinated heterocyclic compounds. Org. Chem. Front..

[81] Park S., Lee S., Kim J.H., Choi W.J., Kim S. (2021). Memory of Chirality in the Asymmetric Synthesis of Piperidines with Vicinal Stereocenters by Intramolecular Sn2′ Reaction. Chem.–Asian J..

[82] Fuji K., Kawabata T. (1998). Memory of Chirality—A New Principle in Enolate Chemistry. Chem.–A Eur. J..

[83] Kawabata T., Fuji K. (2002). Memory of Chirality: Asymmetric Induction Based on the Dynamic Chirality of Enolates. Topics in Stereochemistry.

[84] Alezra V., Kawabata T. (2016). Recent Progress in Memory Of Chirality (MOC): An Advanced Chiral Pool. Synthesis.

[85] Hardwick T., Ahmed N. (2018). Memory of Chirality as a Prominent Pathway for the Synthesis of Natural Products through Chiral Intermediates. ChemistryOpen.

[86] Liu T., Yan N., Zhao H., Wang Z.-X., Hu X.-G. (2018). Synthesis and antibacterial activity of 6(R)- and 6(S)-fluoropenibruguieramine As: Fluorine as a probe for testing the powerfulness of memory of chirality (MOC). J. Fluor. Chem..

[87] Mambrini A., Gori D., Guillot R., Kouklovsky C., Alezra V. (2018). Oxidative coupling of enolates using memory of chirality: An original enantioselective synthesis of quaternary α-amino acid derivatives. Chem. Commun..

[88] Tan S., Li F., Park S., Kim S. (2019). Carbocyclization of Heterosubstituted Alkynes via the Memory of Chirality: Access to Cα-Substituted Proline Derivatives. J. Org. Chem..

[89] Tan S., Li F., Park S., Kim S. (2019). Memory of Chirality in Bromoalkyne Carbocyclization: Applications in Asymmetric Total Synthesis of Hasubanan Alkaloids. Org. Lett..

[90] Li K., Li M.-L., Zhang Q., Zhu S.-F., Zhou Q.-L. (2018). Highly Enantioselective Nickel-Catalyzed Intramolecular Hydroalkenylation of N- and O-Tethered 1,6-Dienes To Form Six-Membered Heterocycles. J. Am. Chem. Soc..

[91] Mori K., Isogai R., Kamei Y., Yamanaka M., Akiyama T. (2018). Chiral Magnesium Bisphosphate-Catalyzed Asymmetric Double C(sp3)–H Bond Functionalization Based on Sequential Hydride Shift/Cyclization Process. J. Am. Chem. Soc..

[92] Cui Q., Liao W., Tian Z.-Y., Li Q., Yu Z.-X. (2019). Rh-Catalyzed Cycloisomerization of 1,7-Ene-Dienes to Synthesize trans-Divinylpiperidines: A Formal Intramolecular Addition Reaction of Allylic C–H Bond into Dienes. Org. Lett..

[93] Liu W., Zhou P., Lang J., Dong S., Liu X., Feng X. (2019). A nickel(ii)-catalyzed asymmetric intramolecular Alder-ene reaction of 1,7-dienes. Chem. Commun..

[94] Shcherbakova V., Dibchak D., Snisarenko M., Skalenko Y., Denisenko A.V., Kuznetsova A.S., Mykhailiuk P.K. (2021). Bicyclic Piperidines via [2 + 2] Photocycloaddition. J. Org. Chem..

[95] Borah M., Saikia A.K. (2018). FeCl3-Mediated Carbenium Ion-Induced Intramolecular Cyclization of N-Tethered Alkyne-Benzyl Alkanols. ChemistrySelect.

[96] Takahashi K., Fukushima K., Seto M., Togashi A., Arai Y., Tsubuki M., Honda T. (2018). Studies on Instructive Construction of exo-Olefin Terminated Five- and Six-Membered Nitrogen Heterocycles: SmI2-Mediated Intramolecular Cyclization of Haloalkynals. J. Org. Chem..

[97] Ding L., Wu W.-T., Zhang L., You S.-L. (2020). Construction of Spironaphthalenones via Gold-Catalyzed Intramolecular Dearomatization Reaction of β-Naphthol Derivatives. Org. Lett..

[98] Kamimura A., Itaya T., Yoshinaga T., Nozawa R., Kawamoto T., Sumimoto M., Uno H. (2020). Highly Cumulated Radical Cascade Reaction of aza-1,6-Enyenes: Stereoselective Synthesis of exo-Methylene Piperidines. Eur. J. Org. Chem..

[99] Mutra M.R., Dhandabani G.K., Li J., Wang J.-J. (2020). Regio- and chemoselective synthesis of nitrogen-containing heterocycles via the oxidative cascade cyclization of unactivated 1,n-enynes. Chem. Commun..

[100] Gharpure S.J., Vishwakarma D.S., Patel R.K. (2019). TMSOTf mediated ‘5/6-endo-dig’ reductive hydroamination for the stereoselective synthesis of pyrrolidine and piperidine derivatives. Chem. Commun..

[101] Lankelma M., Olivares A.M., de Bruin B. (2019). [Co(TPP)]-Catalyzed Formation of Substituted Piperidines. Chem.–A Eur. J..

[102] Herold S., Bafaluy D., Muñiz K. (2018). Anodic benzylic C(sp3)–H amination: Unified access to pyrrolidines and piperidines. Green Chem..

[103] Bafaluy D., Muñoz-Molina J.M., Funes-Ardoiz I., Herold S., de Aguirre A.J., Zhang H., Maseras F., Belderrain T.R., Pérez P.J., Muñiz K. (2019). Copper-Catalyzed N−F Bond Activation for Uniform Intramolecular C−H Amination Yielding Pyrrolidines and Piperidines. Angew. Chem. Int. Ed..

[104] Jin R.-X., Dai J.-C., Li Y., Wang X.-S. (2021). Copper-Catalyzed Intramolecular Amination of C(sp3)–H Bond of Secondary Amines to Access Azacycles. Org. Lett..

[105] Zhang Z., Zhang X., Nagib D.A. (2019). Chiral Piperidines from Acyclic Amines via Enantioselective, Radical-Mediated δ C–H Cyanation. Chem.

[106] Line N.J. (2019). Practical enantioselective synthesis of (3S, 4R)-3-hydroxypiperidine-4-carboxylic acid. Tetrahedron Lett..

[107] Anderson J.C., Bouvier-Israel E., Rundell C.D., Zhang X. (2021). Asymmetric synthesis of piperidines using the nitro-Mannich reaction☆. Tetrahedron.

[108] Cheng P., Lu H., Zu L. (2021). A Local Desymmetrization Approach to Piperidinyl Acetic Acid γ-Secretase Modulators. J. Org. Chem..

[109] Song Q., Wang S., Lei X., Liu Y., Wen X., Wang Z. (2022). One-Pot Route from Halogenated Amides to Piperidines and Pyrrolidines. Molecules.

[110] Wei D., Netkaew C., Carré V., Darcel C. (2019). Iron-Catalysed Reductive Amination of Carbonyl Derivatives with ω-Amino Fatty Acids to Access Cyclic Amines. ChemSusChem.

[111] Xu P., Zhang M., Ingoglia B., Allais C., Dechert-Schmitt A.-M.R., Singer R.A., Morken J.P. (2021). Construction of Azacycles by Intramolecular Amination of Organoboronates and Organobis(boronates). Org. Lett..

[112] Chamberlain A.E.R., Paterson K.J., Armstrong R.J., Twin H.C., Donohoe T.J. (2020). A hydrogen borrowing annulation strategy for the stereocontrolled synthesis of saturated aza-heterocycles. Chem. Commun..

[113] Larsen M.A., Hennessy E.T., Deem M.C., Lam Y.-H., Saurí J., Sather A.C. (2020). A Modular and Diastereoselective 5 + 1 Cyclization Approach to N-(Hetero)Aryl Piperidines. J. Am. Chem. Soc..

[114] McQueen T.M., Griggs S.D. (2021). Efficient synthesis of novel 2-spiropiperidines including an unprecedented non-symmetrical 2,6-bis-spiropiperidine. Tetrahedron Lett..

[115] Ansari A., Ramapanicker R. (2018). Enantioselective Synthesis of 2-Aminomethyl and 3-Amino Pyrrolidines and Piperidines through 1,2-Diamination of Aldehydes. J. Org. Chem..

[116] Li B., Liu S., Wu M., Lin Q., Deng W., Jiang S., Chen L. (2018). Selective ruthenium-catalyzed double reductive aminations using hydrosilane to access tertiary amines and piperidine derivatives. Tetrahedron Lett..

[117] Rao H.S.P., Poonguzhali E., Muthukumaran J. (2021). Synthesis and conformational studies on 1-aryl-cis-2,6-diphenylpiperidines. J. Mol. Struct..

[118] Ouchakour L., Ábrahámi R.A., Forró E., Haukka M., Fülöp F., Kiss L. (2019). Stereocontrolled Synthesis of Fluorine-Containing Piperidine γ-Amino Acid Derivatives. Eur. J. Org. Chem..

[119] Subba Reddy B.V., Nair P.N., Antony A., Lalli C., Grée R. (2017). The Aza-Prins Reaction in the Synthesis of Natural Products and Analogues. Eur. J. Org. Chem..

[120] Jia Y., Li L., Duan L., Li Y.-M. (2020). The aza-Prins Cyclization of Unfunctionalized Olefins Promoted by NHC-Cu Complex and ZrCl4. Appl. Organomet. Chem..

[121] Kamesu K., Mohan G.V.K., Rajasekhar K. (2020). Synthesis of 4-chloropiperidine derivatives via NbCl5 mediated aza-Prins type cyclization of epoxides and homoallylic amines. Rasayan J. Chem..

[122] Lepovitz L.T., Martin S.F. (2019). Diversity-Oriented Synthesis of Bioactive Azaspirocycles. Tetrahedron.

[123] Goodnough A.M., Sahn J.J., Martin S.F. (2020). Facile entry to substituted 2-arylpiperidines via an aza-Sakurai reaction. Tetrahedron Lett..

[124] Ebule R., Mudshinge S., Nantz M.H., Mashuta M.S., Hammond G.B., Xu B. (2019). A 5 + 1 Protic Acid Assisted Aza-Pummerer Approach for Synthesis of 4-Chloropiperidines from Homoallylic Amines. J. Org. Chem..

[125] La M.T., Kang S., Kim H.-K. (2019). Metal-Free Synthesis of N-Aryl-Substituted Azacycles from Cyclic Ethers Using POCl3. J. Org. Chem..

[126] Yin Z., Zhang Y., Zhang S., Wu X.-F. (2019). Copper-catalyzed intra- and intermolecular carbonylative transformation of remote C(sp3)H bonds in N-fluoro-sulfonamides. J. Catal..

[127] Naito Y., Shida N., Atobe M. (2022). Synthesis of piperidine and pyrrolidine derivatives by electroreductive cyclization of imine with terminal dihaloalkanes in a flow microreactor. Beilstein J. Org. Chem..

[128] Ramesh S., Balakumar R., Rizzo J.R., Zhang T.Y. (2019). Enantiospecific Synthesis of 2-Substituted Piperidine-4-carboxylic Acids from α-Amino Acids. ChemistrySelect.

[129] Cendón B., Font M., Mascareñas J.L., Gulías M. (2020). Palladium-Catalyzed Formal (4 + 2) Cycloaddition between Alkyl Amides and Dienes Initiated by the Activation of C(sp3)–H Bonds. ACS Catal..

[130] Liu M., Zhou L., Shi W., Hu Y., Liao J., Duan Z., Wang W., Wu Y., Zheng B., Guo H. (2021). Phosphine-Catalyzed (4 + 2) Annulation of δ-Sulfonamido-Substituted Enones with 1,1-Dicyanoalkenes: Synthesis of Piperidine Derivatives. Org. Lett..

[131] Huang Y., Liao J., Wang W., Liu H., Guo H. (2020). Synthesis of heterocyclic compounds through nucleophilic phosphine catalysis. Chem. Commun..

[132] Xu X., Doyle M.P. (2014). The [3 + 3]-Cycloaddition Alternative for Heterocycle Syntheses: Catalytically Generated Metalloenolcarbenes as Dipolar Adducts. Acc. Chem. Res..

[133] Chithanna S., Roy A., Yang D.-Y. (2021). Acid-catalyzed, regioselective [3 + 3] annulation of enaminones and α-substituted cinnamic acids: Access to 3,4-dihydropyridones and 2-piperidinones. Org. Biomol. Chem..

[134] Poddar R., Jain A., Kidwai M. (2017). Bis[(l)prolinate-N,O]Zn: A water-soluble and recycle catalyst for various organic transformations. J. Adv. Res..

[135] Fan W., Verrier C., Wang L., Ahmar M., Tan J.-N., Popowycz F., Queneau Y., Rauter A.P., Christensen B.E., Somsák L., Kosma P., Adamo R. (2020). 2-5-(Hydroxymethyl)furfural and 5-(glucosyloxymethyl)furfural in multicomponent reactions. Recent Trends in Carbohydrate Chemistry.

[136] Dömling A., Ugi I. (2000). Multicomponent Reactions with Isocyanides. Angew. Chem. Int. Ed..

[137] Dömling A. (2006). Recent Developments in Isocyanide Based Multicomponent Reactions in Applied Chemistry. Chem. Rev..

[138] Ruijter E., Scheffelaar R., Orru R.V.A. (2011). Multicomponent Reaction Design in the Quest for Molecular Complexity and Diversity. Angew. Chem. Int. Ed..

[139] Sunderhaus J.D., Martin S.F. (2009). Applications of Multicomponent Reactions to the Synthesis of Diverse Heterocyclic Scaffolds. Chem.–A Eur. J..

[140] Filho J.F.A., Lemos B.C., de Souza A.S., Pinheiro S., Greco S.J. (2017). Multicomponent Mannich reactions: General aspects, methodologies and applications. Tetrahedron.

[141] Touré B.B., Hall D.G. (2009). Natural Product Synthesis Using Multicomponent Reaction Strategies. Chem. Rev..

[142] Guareschi I. (1897). New investigations about the synthesis of Pyridine compounds and the reaction of Hantzsch. Estr. aus Atti R. Accad. Torino.

[143] Guareschi I. (1899). About Dicyano Dioxypyridine. Estr. aus Atti R. Accad. Torino.

[144] Minozzi A. (1900). Synthesis of glutaric and trimethylene derivatives. Gazz. Chim. Ital..

[145] Kangani M., Hazeri N., Yazdani-Elah-Abadi A., Maghsoodlou M.-T. (2018). Lactic Acid: An Efficient and Green Catalyst for the One-Pot Five-Components Synthesis of Highly Substituted Piperidines. Polycycl. Aromat. Compd..

[146] Sharma G.V.R., Naidu A.A. (2018). Synthesis of Highly Functionalized Piperidines by One-Pot Multicomponent Reaction Using Boron Trifluoride Etherate. Chem. Sci. Trans..

[147] Vereshchagin A.N., Karpenko K.A., Iliyasov T.M., Elinson M.N., Dorofeeva E.O., Fakhrutdinov A.N., Egorov M.P. (2018). Diastereoselective multicomponent synthesis of (4RS,6SR)-4,6-diaryl-5,5-dicyano-2-methyl-1,4,5,6-tetrahydropyridine-3-carboxylates. Russ. Chem. Bull..

[148] Mohamadpour F. (2020). Green and Convenient One-Pot Access to Polyfunctionalized Piperidine Scaffolds via Glutamic Acid Catalyzed Knoevenagel- Intramolecular [4 + 2] aza-Diels-Alder Imin-Based Multi-Component Reaction Under Ambient Temperature. Polycycl. Aromat. Compd..

[149] Wu L., Yan S., Wang W., Li Y. (2020). Multicomponent reaction for the synthesis of highly functionalized piperidine scaffolds catalyzed by TMSI. Res. Chem. Intermed..

[150] Ghamari Kargar P., Bagherzade G. (2021). Robust, highly active, and stable supported Co(ii) nanoparticles on magnetic cellulose nanofiber-functionalized for the multi-component reactions of piperidines and alcohol oxidation. RSC Adv..

[151] Vereshchagin A.N., Iliyasov T.M., Karpenko K.A., Smirnov V.A., Ushakov I.E., Elinson M.N. (2021). Highly diastereoselective four-component synthesis of polysubstituted 1,4,5,6-tetrahydropyridines. Chem. Heterocycl. Compd..

[152] Vettukattil U., Govindan A., James K., Anilkumar A., Krishnapillai S. (2021). Efficient synthesis of piperidine derivatives using dendrimer based catalytical pockets. J. Heterocycl. Chem..

[153] Gadge D.D., Kulkarni P.S. (2022). 5-Sulfosalicylic acid an organocatalyst for the synthesis of highly functionalized piperidines through the multicomponent reaction. J. Heterocycl. Chem..

[154] Ryzhkova Y.E., Elinson M.N. (2022). Multicomponent synthesis of 1,4-dihydropyridine-3,5-dicarbonitriles. Arkivoc.

[155] Khatun R., Biswas S., Ghosh S., Islam S.M. (2018). Polymer-anchored [Fe(III)Azo] complex: An efficient reusable catalyst for oxidative bromination and multi-components reaction for the synthesis of spiropiperidine derivatives. J. Organomet. Chem..

[156] Laskar K., Farhan M., Ahmad A. (2021). Yb/Chitosan Catalyzed Synthesis of Highly Substituted Piperidine Derivatives for Potential Nuclease Activity and DNA Binding Study. Curr. Pharm. Des..

[157] Heravi M.M., Zadsirjan V., Dehghani M., Ahmadi T. (2018). Towards click chemistry: Multicomponent reactions via combinations of name reactions. Tetrahedron.

[158] Vereshchagin A.N., Karpenko K.A., Elinson M.N., Dorofeeva E.O., Goloveshkin A.S., Egorov M.P. (2018). Pseudo six-component stereoselective synthesis of 2,4,6-triaryl-3,3,5,5-tetracyanopiperidines. Mendeleev Commun..

[159] Vereshchagin A.N., Karpenko K.A., Elinson M.N., Goloveshkin A.S., Ushakov I.E., Egorov M.P. (2018). Four-component stereoselective synthesis of tetracyano-substituted piperidines. Res. Chem. Intermed..

[160] Vereshchagin A.N., Karpenko K.A., Elinson M.N., Gorbunov S.V., Anisina Y.E., Egorov M.P. (2018). Stereoselective multicomponent synthesis of (2RS,6SR)-2,6-diaryl-3,3,5,5-tetracyanopiperidines. Russ. Chem. Bull..

[161] Vereshchagin A.N., Karpenko K.A., Elinson M.N., Gorbunov S.V., Gordeeva A.M., Proshin P.I., Goloveshkin A.S., Egorov M.P. (2018). Stereoselective one-pot synthesis of polycyanosubstituted piperidines. Mon. Für Chem.–Chem. Mon..

[162] Vereshchagin A.N., Iliyasov T.M., Karpenko K.A., Akchurin R.N., Minyaev M.E. (2022). Tetrahydropyridines & rsquo; Stereoselective Formation, How Lockdown Assisted in the Identification of the Features of Its Mechanism. Molecules.

[163] Karpenko K.A., Iliyasov T.M., Fakhrutdinov A.N., Akulinin A.S., Elinson M.N., Vereshchagin A.N. (2022). Study on formation mechanism of (4RS,6SR)-4,6-diaryl-5,5-dicyano-2-methyl-1,4,5,6-tetrahydropyridine-3-carboxylic esters. Russ. Chem. Bull..

[164] José Climent M., Corma A., Iborra S. (2012). Homogeneous and heterogeneous catalysts for multicomponent reactions. RSC Adv..

[165] Martin K.S., Di Maso M.J., Fettinger J.C., Shaw J.T. (2013). Synthesis of a Library of “Lead-Like” γ-Lactams by a One Pot, Four-Component Reaction. ACS Comb. Sci..

[166] Zhou Z., Liu H., Li Y., Liu J., Li Y., Liu J., Yao J., Wang C. (2013). Novel Synthesis of Substituted Furo[3,2-c]chromen-4-ones via Four-component Reaction from Substituted Nitrostyrenes, Aromatic Aldehydes, Coumarins, and Ammonium Acetate. ACS Comb. Sci..

[167] Kumar D., Kommi D.N., Bollineni N., Patel A.R., Chakraborti A.K. (2012). Catalytic procedures for multicomponent synthesis of imidazoles: Selectivity control during the competitive formation of tri- and tetrasubstituted imidazoles. Green Chem..

[168] Moosavi-Zare A.R., Asgari Z., Zare A., Zolfigol M.A., Shekouhy M. (2014). One pot synthesis of 1,2,4,5-tetrasubstituted-imidazoles catalyzed by trityl chloride in neutral media. RSC Adv..

[169] Chundawat T.S., Sharma N., Kumari P., Bhagat S. (2015). Microwave-Assisted Nickel-Catalyzed One-Pot Synthesis of 2,4,5-Trisubstituted Imidazoles. Synlett.

[170] Niknam K., Deris A., Naeimi F., Majleci F. (2011). Synthesis of 1,2,4,5-tetrasubstituted imidazoles using silica-bonded propylpiperazine N-sulfamic acid as a recyclable solid acid catalyst. Tetrahedron Lett..

[171] MaGee D.I., Bahramnejad M., Dabiri M. (2013). Highly efficient and eco-friendly synthesis of 2-alkyl and 2-aryl-4,5-diphenyl-1H-imidazoles under mild conditions. Tetrahedron Lett..

[172] Adib M., Ayashi N., Mirzaei P. (2015). An Efficient Synthesis of 2,4,6-Triarylpyridines by Use of Benzyl Halides under Neat Conditions. Synlett.

[173] Pagadala R., Kommidi D.R., Rana S., Maddila S., Moodley B., Koorbanally N.A., Jonnalagadda S.B. (2015). Multicomponent synthesis of pyridines via diamine functionalized mesoporous ZrO2 domino intramolecular tandem Michael type addition. RSC Adv..

[174] Maleki A., Rahimi R., Maleki S., Hamidi N. (2014). Synthesis and characterization of magnetic bromochromate hybrid nanomaterials with triphenylphosphine surface-modified iron oxide nanoparticles and their catalytic application in multicomponent reactions. RSC Adv..

[175] Dam B., Nandi S., Pal A.K. (2014). An efficient ‘on-water’ synthesis of 1,4-dihydropyridines using Fe3O4@SiO2 nanoparticles as a reusable catalyst. Tetrahedron Lett..

[176] Singh M.S., Chowdhury S. (2012). Recent developments in solvent-free multicomponent reactions: A perfect synergy for eco-compatible organic synthesis. RSC Adv..

[177] Jagadale M.B., Salunkhe R.S., Rajmane M.M., Dhanavade M.J., Sonawane K.D., Rashinkar G.S. (2018). Water-Mediated Synthesis of Anthelmintic Piperidinols and Their Molecular Docking Studies. ChemistrySelect.

[178] Van Beek W.E., Gadde K., Tehrani K.A. (2018). The Use of Calcium Carbide as Acetylene Source in a Three-Component Coupling with ω-Chlorinated Ketones and Primary Amines. Chem.–A Eur. J..

[179] Vereshchagin A.N., Karpenko K.A., Elinson M.N., Goloveshkin A.S., Dorofeeva E.O., Egorov M.P. (2020). Highly diastereoselective four-component synthesis of polysubstituted 2-piperidinones with three and four stereogenic centers. Res. Chem. Intermed..

[180] Vereshchagin A.N., Karpenko K.A., Elinson M.N., Minaeva A.P., Goloveshkin A.S., Hansford K.A., Egorov M.P. (2020). One-pot five-component high diastereoselective synthesis of polysubstituted 2-piperidinones from aromatic aldehydes, nitriles, dialkyl malonates and ammonium acetate. Mol. Divers..

[181] Savithiri S., Bharanidharan S., Sugumar P., Rajeevgandhi C., Indhira M. (2021). Synthesis, spectral, stereochemical, biological, molecular docking and DFT studies of 3-alkyl/3,5-dialkyl-2r,6c-di(naphthyl)piperidin-4-one picrates derivatives. J. Mol. Struct..

[182] Hemalatha K., Ilangeswaran D. (2020). One pot synthesis of 2,6-bis(2/4-hydroxyphenyl)piperidin-4-one derivatives using greener deep eutectic solvent media and their characterization. Mater. Today Proc..

[183] Kong L., Huang R., He H., Fan Y., Lin J., Yan S. (2020). Multi-component solvent-free cascade reaction of 2-cyanoacetamides: Regioselective synthesis of pyridin-2-ones bearing quaternary centers. Green Chem..

[184] Vardanyan R., Vardanyan R. (2017). Chapter 10–Classes of Piperidine-Based Drugs. Piperidine-Based Drug Discovery.

[185] Goel P., Alam O., Naim M.J., Nawaz F., Iqbal M., Alam M.I. (2018). Recent advancement of piperidine moiety in treatment of cancer- A review. Eur. J. Med. Chem..

[186] Charalambous A., Schwarzbich M.-A., Witzens-Harig M., Martens U.M. (2018). Ibrutinib. Small Molecules in Hematology.

[187] Milling R.V., Grimm D., Krüger M., Grosse J., Kopp S., Bauer J., Infanger M., Wehland M. (2018). Pazopanib, Cabozantinib, and Vandetanib in the Treatment of Progressive Medullary Thyroid Cancer with a Special Focus on the Adverse Effects on Hypertension. Int. J. Mol. Sci..

[188] Coricello A., Mesiti F., Lupia A., Maruca A., Alcaro S. (2020). Inside Perspective of the Synthetic and Computational Toolbox of JAK Inhibitors: Recent Updates. Molecules.

[189] Shaw V., Srivastava S., Srivastava S.K. (2021). Repurposing antipsychotics of the diphenylbutylpiperidine class for cancer therapy. Semin. Cancer Biol..

[190] Ezelarab H.A.A., Abbas S.H., Hassan H.A., Abuo-Rahma G.E.-D.A. (2018). Recent updates of fluoroquinolones as antibacterial agents. Arch. Der Pharm..

[191] Ye N., Qin W., Tian S., Xu Q., Wold E.A., Zhou J., Zhen X.-C. (2020). Small Molecules Selectively Targeting Sigma-1 Receptor for the Treatment of Neurological Diseases. J. Med. Chem..

[192] Friedman J.H. (2018). Pharmacological interventions for psychosis in Parkinson’s disease patients. Expert Opin. Pharmacother..

[193] Kantrowitz J.T. (2020). Targeting Serotonin 5-HT2A Receptors to Better Treat Schizophrenia: Rationale and Current Approaches. CNS Drugs.

[194] Rk M., Begum S., Begum A., Koganti B. (2018). Antioxidant potential of piperidine containing compounds–A short review. Asian J. Pharm. Clin. Res..

[195] Lakstygal A.M., Kolesnikova T.O., Khatsko S.L., Zabegalov K.N., Volgin A.D., Demin K.A., Shevyrin V.A., Wappler-Guzzetta E.A., Kalueff A.V. (2019). DARK Classics in Chemical Neuroscience: Atropine, Scopolamine, and Other Anticholinergic Deliriant Hallucinogens. ACS Chem. Neurosci..

[196] Vagge A., Ferro Desideri L., Nucci P., Serafino M., Giannaccare G., Traverso C.E. (2018). Prevention of Progression in Myopia: A Systematic Review. Diseases.

[197] Devereaux A.L., Mercer S.L., Cunningham C.W. (2018). DARK Classics in Chemical Neuroscience: Morphine. ACS Chem. Neurosci..

[198] Cavalli E., Mammana S., Nicoletti F., Bramanti P., Mazzon E. (2019). The neuropathic pain: An overview of the current treatment and future therapeutic approaches. Int. J. Immunopathol. Pharmacol..

[199] Shityakov S., Bigdelian E., Hussein A.A., Hussain M.B., Tripathi Y.C., Khan M.U., Shariati M.A. (2019). Phytochemical and pharmacological attributes of piperine: A bioactive ingredient of black pepper. Eur. J. Med. Chem..

[200] Manayi A., Nabavi M.S., Setzer N.W., Jafari S. (2018). Piperine as a Potential Anti-cancer Agent: A Review on Preclinical Studies. Curr. Med. Chem..

[201] Smilkov K., Ackova G.D., Cvetkovski A., Ruskovska T., Vidovic B., Atalay M. (2019). Piperine: Old Spice and New Nutraceutical?. Curr. Pharm. Des..

[202] Afreen, Salahuddin, Mazumder A., Joshi S., Kumar R., Yar S.M., Ahsan J.M. (2021). Insight into the Isolation, Synthesis, and Structure-Activity Relationship of Piperine Derivatives for the Development of New Compounds: Recent Updates. Curr. Top. Med. Chem..

[203] Haq I.-U., Imran M., Nadeem M., Tufail T., Gondal T.A., Mubarak M.S. (2021). Piperine: A review of its biological effects. Phytother. Res..

[204] Cheng Y., Rauf A., Pan X. (2022). Research Progress on the Natural Product Aloperine and Its Derivatives. Mini-Rev. Med. Chem..

[205] Singh L., Upadhyay K.A., Dixit P., Singh A., Yadav D., Chhavi A., Konar S., Srivastava P.R., Pandey S., Devkota P.H. (2022). A Review of Chemistry and Pharmacology of Piperidine Alkaloids of Pinus and Related Genera. Curr. Pharm. Biotechnol..

[206] Ward R.A., Fawell S., Floc′h N., Flemington V., McKerrecher D., Smith P.D. (2021). Challenges and Opportunities in Cancer Drug Resistance. Chem. Rev..

[207] Arumugam N., Almansour A.I., Kumar R.S., Al-thamili D.M., Periyasami G., Periasamy V.S., Athinarayanan J., Alshatwi A.A., Mahalingam S.M., Menéndez J.C. (2018). Regio and stereoselective synthesis of anticancer spirooxindolopyrrolidine embedded piperidone heterocyclic hybrids derived from one-pot cascade protocol. Chem. Cent. J..

[208] Lovering F., Bikker J., Humblet C. (2009). Escape from Flatland: Increasing Saturation as an Approach to Improving Clinical Success. J. Med. Chem..

[209] Lovering F. (2013). Escape from Flatland 2: Complexity and promiscuity. MedChemComm.

[210] Harmata A.S., Roldan B.J., Stephenson C.R.J. (2023). Formal Cycloadditions Driven by the Homolytic Opening of Strained, Saturated Ring Systems. Angew. Chem. Int. Ed..

[211] Dayal N., Wang M., Sintim H.O. (2020). HSD1787, a Tetrahydro-3H-Pyrazolo[4,3-f]Quinoline Compound Synthesized via Povarov Reaction, Potently Inhibits Proliferation of Cancer Cell Lines at Nanomolar Concentrations. ACS Omega.

[212] Irie T., Asami T., Sawa A., Uno Y., Taniyama C., Funakoshi Y., Masai H., Sawa M. (2021). Discovery of AS-0141, a Potent and Selective Inhibitor of CDC7 Kinase for the Treatment of Solid Cancers. J. Med. Chem..

[213] Kuznetcova I., Ostojić M., Gligorijević N., Aranđelović S., Arion V.B. (2022). Enriching Chemical Space of Bioactive Scaffolds by New Ring Systems: Benzazocines and Their Metal Complexes as Potential Anticancer Drugs. Inorg. Chem..

[214] Yin D.-L., Liang Y.-J., Zheng T.-S., Song R.-P., Wang J.-B., Sun B.-S., Pan S.-H., Qu L.-D., Liu J.-R., Jiang H.-C. (2016). EF24 inhibits tumor growth and metastasis via suppressing NF-kappaB dependent pathways in human cholangiocarcinoma. Sci. Rep..

[215] He Y., Li W., Hu G., Sun H., Kong Q. (2018). Bioactivities of EF24, a Novel Curcumin Analog: A Review. Front. Oncol..

[216] Bazzaro M., Linder S. (2020). Dienone Compounds: Targets and Pharmacological Responses. J. Med. Chem..

[217] Khurana N., Dodhiawala P.B., Bulle A., Lim K.-H. (2020). Deciphering the Role of Innate Immune NF-ĸB Pathway in Pancreatic Cancer. Cancers.

[218] Liu S., Jiang Y., Yan R., Li Z., Wan S., Zhang T., Wu X., Hou J., Zhu Z., Tian Y. (2019). Design, synthesis and biological evaluations of 2-amino-4-(1-piperidine) pyridine derivatives as novel anti crizotinib-resistant ALK/ROS1 dual inhibitors. Eur. J. Med. Chem..

[219] Golding B., Luu A., Jones R., Viloria-Petit A.M. (2018). The function and therapeutic targeting of anaplastic lymphoma kinase (ALK) in non-small cell lung cancer (NSCLC). Mol. Cancer.

[220] Kong X., Pan P., Sun H., Xia H., Wang X., Li Y., Hou T. (2019). Drug Discovery Targeting Anaplastic Lymphoma Kinase (ALK). J. Med. Chem..

[221] Du X., Shao Y., Qin H.-F., Tai Y.-H., Gao H.-J. (2018). ALK-rearrangement in non-small-cell lung cancer (NSCLC). Thorac. Cancer.

[222] Guaitoli G., Bertolini F., Bettelli S., Manfredini S., Maur M., Trudu L., Aramini B., Masciale V., Grisendi G., Dominici M. (2021). Deepening the Knowledge of ROS1 Rearrangements in Non-Small Cell Lung Cancer: Diagnosis, Treatment, Resistance and Concomitant Alterations. Int. J. Mol. Sci..

[223] Cui J.J., Tran-Dubé M., Shen H., Nambu M., Kung P.-P., Pairish M., Jia L., Meng J., Funk L., Botrous I. (2011). Structure Based Drug Design of Crizotinib (PF-02341066), a Potent and Selective Dual Inhibitor of Mesenchymal–Epithelial Transition Factor (c-MET) Kinase and Anaplastic Lymphoma Kinase (ALK). J. Med. Chem..

[224] Radaram B., Pisaneschi F., Rao Y., Yang P., Piwnica-Worms D., Alauddin M.M. (2019). Novel derivatives of anaplastic lymphoma kinase inhibitors: Synthesis, radiolabeling, and preliminary biological studies of fluoroethyl analogues of crizotinib, alectinib, and ceritinib. Eur. J. Med. Chem..

[225] Moi D., Nocentini A., Deplano A., Osman S.M., AlOthman Z.A., Piras V., Balboni G., Supuran C.T., Onnis V. (2020). Appliance of the piperidinyl-hydrazidoureido linker to benzenesulfonamide compounds: Synthesis, in vitro and in silico evaluation of potent carbonic anhydrase II, IX and XII inhibitors. Bioorganic Chem..

[226] Mishra C.B., Tiwari M., Supuran C.T. (2020). Progress in the development of human carbonic anhydrase inhibitors and their pharmacological applications: Where are we today?. Med. Res. Rev..

[227] Krasavin M., Kalinin S., Sharonova T., Supuran C.T. (2020). Inhibitory activity against carbonic anhydrase IX and XII as a candidate selection criterion in the development of new anticancer agents. J. Enzym. Inhib. Med. Chem..

[228] McDonald P.C., Chia S., Bedard P.L., Chu Q., Lyle M., Tang L., Singh M., Zhang Z., Supuran C.T., Renouf D.J. (2020). A Phase 1 Study of SLC-0111, a Novel Inhibitor of Carbonic Anhydrase IX, in Patients With Advanced Solid Tumors. Am. J. Clin. Oncol..

[229] Ciccone V., Filippelli A., Angeli A., Supuran C.T., Morbidelli L. (2020). Pharmacological Inhibition of CA-IX Impairs Tumor Cell Proliferation, Migration and Invasiveness. Int. J. Mol. Sci..

[230] Bononi G., Tonarini G., Poli G., Barravecchia I., Caligiuri I., Macchia M., Rizzolio F., Demontis G.C., Minutolo F., Granchi C. (2021). Monoacylglycerol lipase (MAGL) inhibitors based on a diphenylsulfide-benzoylpiperidine scaffold. Eur. J. Med. Chem..

[231] Granchi C., Bononi G., Ferrisi R., Gori E., Mantini G., Glasmacher S., Poli G., Palazzolo S., Caligiuri I., Rizzolio F. (2021). Design, synthesis and biological evaluation of second-generation benzoylpiperidine derivatives as reversible monoacylglycerol lipase (MAGL) inhibitors. Eur. J. Med. Chem..

[232] Gil-Ordóñez A., Martín-Fontecha M., Ortega-Gutiérrez S., López-Rodríguez M.L. (2018). Monoacylglycerol lipase (MAGL) as a promising therapeutic target. Biochem. Pharmacol..

[233] Chitti S., Pulya S., Nandikolla A., Patel T.K., Karan Kumar B., Murugesan S., Ghosh B., Sekhar K.V.G.C. (2021). Design, synthesis and biological evaluation of 7–(5–((substituted–amino)-methyl)-thiophen–2–yl)-spiro-[chroman–2,4′–piperidin]–4–one hydrochloride analogues as anticancer agents. Bioorganic Chem..

[234] Karmacharya U., Chaudhary P., Lim D., Dahal S., Awasthi B.P., Park H.D., Kim J.-A., Jeong B.-S. (2021). Synthesis and anticancer evaluation of 6-azacyclonol-2,4,6-trimethylpyridin-3-ol derivatives: M3 muscarinic acetylcholine receptor-mediated anticancer activity of a cyclohexyl derivative in androgen-refractory prostate cancer. Bioorganic Chem..

[235] Felton J., Hu S., Raufman J.-P. (2018). Targeting M3 Muscarinic Receptors for Colon Cancer Therapy. Curr. Mol. Pharmacol..

[236] Tolaymat M., Larabee S.M., Hu S., Xie G., Raufman J.-P. (2019). The Role of M3 Muscarinic Receptor Ligand-Induced Kinase Signaling in Colon Cancer Progression. Cancers.

[237] Ali O., Tolaymat M., Hu S., Xie G., Raufman J.-P. (2021). Overcoming Obstacles to Targeting Muscarinic Receptor Signaling in Colorectal Cancer. Int. J. Mol. Sci..

[238] Scheltens P., De Strooper B., Kivipelto M., Holstege H., Chételat G., Teunissen C.E., Cummings J., van der Flier W.M. (2021). Alzheimer′s disease. Lancet.

[239] Hampel H., Mesulam M.M., Cuello A.C., Khachaturian A.S., Vergallo A., Farlow M.R., Snyder P.J., Giacobini E., Khachaturian Z.S., Cholinergic System Working G. (2019). Revisiting the Cholinergic Hypothesis in Alzheimer’s Disease: Emerging Evidence from Translational and Clinical Research. J. Prev. Alzheimer’s Dis..

[240] Marucci G., Buccioni M., Ben D.D., Lambertucci C., Volpini R., Amenta F. (2021). Efficacy of acetylcholinesterase inhibitors in Alzheimer′s disease. Neuropharmacology.

[241] Stanciu G.D., Luca A., Rusu R.N., Bild V., Beschea Chiriac S.I., Solcan C., Bild W., Ababei D.C. (2020). Alzheimer’s Disease Pharmacotherapy in Relation to Cholinergic System Involvement. Biomolecules.

[242] Chierrito T.P.C., Pedersoli-Mantoani S., Roca C., Sebastian-Pérez V., Martínez-Gonzalez L., Pérez D.I., Perez C., Canales A., Cañada F.J., Campillo N.E. (2018). Chameleon-like behavior of indolylpiperidines in complex with cholinesterases targets: Potent butyrylcholinesterase inhibitors. Eur. J. Med. Chem..

[243] Cai R., Wang L.-N., Fan J.-J., Geng S.-Q., Liu Y.-M. (2019). New 4-N-phenylaminoquinoline derivatives as antioxidant, metal chelating and cholinesterase inhibitors for Alzheimer’s disease. Bioorganic Chem..

[244] Zhu J., Wang L.-N., Cai R., Geng S.-Q., Dong Y.-F., Liu Y.-M. (2019). Design, synthesis, evaluation and molecular modeling study of 4-N-phenylaminoquinolines for Alzheimer disease treatment. Bioorganic Med. Chem. Lett..

[245] Košak U., Brus B., Knez D., Žakelj S., Trontelj J., Pišlar A., Šink R., Jukič M., Živin M., Podkowa A. (2018). The Magic of Crystal Structure-Based Inhibitor Optimization: Development of a Butyrylcholinesterase Inhibitor with Picomolar Affinity and in Vivo Activity. J. Med. Chem..

[246] Košak U., Strašek N., Knez D., Jukič M., Žakelj S., Zahirović A., Pišlar A., Brazzolotto X., Nachon F., Kos J. (2020). N-alkylpiperidine carbamates as potential anti-Alzheimer’s agents. Eur. J. Med. Chem..

[247] Wichur T., Więckowska A., Więckowski K., Godyń J., Jończyk J., Valdivieso Á.d.R., Panek D., Pasieka A., Sabaté R., Knez D. (2020). 1-Benzylpyrrolidine-3-amine-based BuChE inhibitors with anti-aggregating, antioxidant and metal-chelating properties as multifunctional agents against Alzheimer’s disease. Eur. J. Med. Chem..

[248] Prati F., Bottegoni G., Bolognesi M.L., Cavalli A. (2018). BACE-1 Inhibitors: From Recent Single-Target Molecules to Multitarget Compounds for Alzheimer’s Disease. J. Med. Chem..

[249] Gehlot P., Kumar S., Kumar Vyas V., Singh Choudhary B., Sharma M., Malik R. (2022). Guanidine-based β amyloid precursor protein cleavage enzyme 1 (BACE-1) inhibitors for the Alzheimer′s disease (AD): A review. Bioorganic Med. Chem..

[250] Frisoni G.B., Altomare D., Thal D.R., Ribaldi F., van der Kant R., Ossenkoppele R., Blennow K., Cummings J., van Duijn C., Nilsson P.M. (2022). The probabilistic model of Alzheimer disease: The amyloid hypothesis revised. Nat. Rev. Neurosci..

[251] Sugimoto H., Yamanish Y., Iimura Y., Kawakami Y. (2000). Donepezil Hydrochloride (E2020) and Other Acetylcholinesterase Inhibitors. Curr. Med. Chem..

[252] El-Sayed N.A.-E., Farag A.E.-S., Ezzat M.A.F., Akincioglu H., Gülçin İ., Abou-Seri S.M. (2019). Design, synthesis, in vitro and in vivo evaluation of novel pyrrolizine-based compounds with potential activity as cholinesterase inhibitors and anti-Alzheimer′s agents. Bioorganic Chem..

[253] Sharma P., Tripathi A., Tripathi P.N., Prajapati S.K., Seth A., Tripathi M.K., Srivastava P., Tiwari V., Krishnamurthy S., Shrivastava S.K. (2019). Design and development of multitarget-directed N-Benzylpiperidine analogs as potential candidates for the treatment of Alzheimer′s disease. Eur. J. Med. Chem..

[254] van Greunen D.G., Johan van der Westhuizen C., Cordier W., Nell M., Stander A., Steenkamp V., Panayides J.-L., Riley D.L. (2019). Novel N-benzylpiperidine carboxamide derivatives as potential cholinesterase inhibitors for the treatment of Alzheimer′s disease. Eur. J. Med. Chem..

[255] Sadeghian B., Sakhteman A., Faghih Z., Nadri H., Edraki N., Iraji A., Sadeghian I., Rezaei Z. (2020). Design, synthesis and biological activity evaluation of novel carbazole-benzylpiperidine hybrids as potential anti Alzheimer agents. J. Mol. Struct..

[256] Chowdhury S.R., Gu J., Hu Y., Wang J., Lei S., Tavallaie M.S., Lam C., Lu D., Jiang F., Fu L. (2021). Synthesis, biological evaluation and molecular modeling of benzofuran piperidine derivatives as Aβ antiaggregant. Eur. J. Med. Chem..

[257] Wang K., Shi J., Zhou Y., He Y., Mi J., Yang J., Liu S., Tang X., Liu W., Tan Z. (2021). Design, synthesis and evaluation of cinnamic acid hybrids as multi-target-directed agents for the treatment of Alzheimer′s disease. Bioorganic Chem..

[258] Liu W., Wu L., Liu W., Tian L., Chen H., Wu Z., Wang N., Liu X., Qiu J., Feng X. (2022). Design, synthesis and biological evaluation of novel coumarin derivatives as multifunctional ligands for the treatment of Alzheimer′s disease. Eur. J. Med. Chem..

[259] Vereshchagin A.N., Frolov N.A., Egorova K.S., Seitkalieva M.M., Ananikov V.P. (2021). Quaternary Ammonium Compounds (QACs) and Ionic Liquids (ILs) as Biocides: From Simple Antiseptics to Tunable Antimicrobials. Int. J. Mol. Sci..

[260] Li B., Wang K., Zhang R., Li B., Shen Y., Ji Q. (2019). Design, synthesis and biological evaluation of novel diazaspiro[4.5]decan-1-one derivatives as potential chitin synthase inhibitors and antifungal agents. Eur. J. Med. Chem..

[261] Krauß J., Müller C., Klimt M., Valero L.J., Martínez J.F., Müller M., Bartel K., Binder U., Bracher F. (2021). Synthesis, Biological Evaluation, and Structure & ndash; Activity Relationships of 4-Aminopiperidines as Novel Antifungal Agents Targeting Ergosterol Biosynthesis. Molecules.

[262] Rodrigues Marcio L. (2018). The Multifunctional Fungal Ergosterol. Mbio.

[263] Hong G., Li W., Mao L., Wang J., Liu T. (2022). Synthesis and antibacterial activity evaluation of N (7) position-modified balofloxacins. Front. Chem..

[264] Karoutzou O., Benaki D., Papanastasiou I., Vocat A., Cole S.T. (2019). Synthesis of New Indole and Adamantane Amido Derivatives with Pharmacological Interest. ChemistrySelect.

[265] de Castro S., Ginex T., Vanderlinden E., Laporte M., Stevaert A., Cumella J., Gago F., Camarasa M.J., Luque F.J., Naesens L. (2020). N-benzyl 4,4-disubstituted piperidines as a potent class of influenza H1N1 virus inhibitors showing a novel mechanism of hemagglutinin fusion peptide interaction. Eur. J. Med. Chem..

[266] Nandini Asha R., Ravindran Durai Nayagam B., Bhuvanesh N. (2021). Synthesis, molecular docking, and in silico ADMET studies of 4-benzyl-1-(2,4,6-trimethyl-benzyl)-piperidine: Potential Inhibitor of SARS-CoV2. Bioorganic Chem..

[267] Lepovitz L.T., Meis A.R., Thomas S.M., Wiedeman J., Pham A., Mensa-Wilmot K., Martin S.F. (2020). Design, synthesis, and evaluation of novel anti-trypanosomal compounds. Tetrahedron.

[268] Van de Walle T., Boone M., Van Puyvelde J., Combrinck J., Smith P.J., Chibale K., Mangelinckx S., D′hooghe M. (2020). Synthesis and biological evaluation of novel quinoline-piperidine scaffolds as antiplasmodium agents. Eur. J. Med. Chem..

[269] Seck R., Gassama A., Cojean S., Cavé C. (2020). Synthesis and Antimalarial Activity of 1,4-Disubstituted Piperidine Derivatives. Molecules.

[270] Colloca L., Ludman T., Bouhassira D., Baron R., Dickenson A.H., Yarnitsky D., Freeman R., Truini A., Attal N., Finnerup N.B. (2017). Neuropathic pain. Nat. Rev. Dis. Primers.

[271] Valentino R.J., Volkow N.D. (2018). Untangling the complexity of opioid receptor function. Neuropsychopharmacology.

[272] Günther T., Dasgupta P., Mann A., Miess E., Kliewer A., Fritzwanker S., Steinborn R., Schulz S. (2018). Targeting multiple opioid receptors–improved analgesics with reduced side effects?. Br. J. Pharmacol..

[273] Armenian P., Vo K.T., Barr-Walker J., Lynch K.L. (2018). Fentanyl, fentanyl analogs and novel synthetic opioids: A comprehensive review. Neuropharmacology.

[274] Nami M., Salehi P., Dabiri M., Bararjanian M., Gharaghani S., Khoramjouy M., Al-Harrasi A., Faizi M. (2018). Synthesis of novel norsufentanil analogs via a four-component Ugi reaction and in vivo, docking, and QSAR studies of their analgesic activity. Chem. Biol. Drug Des..

[275] Hsu F.-L., Walz A.J., Myslinski J.M., Kong L., Feasel M.G., Goralski T.D.P., Rose T., Cooper N.J., Roughley N., Timperley C.M. (2019). Synthesis and μ-Opioid Activity of the Primary Metabolites of Carfentanil. ACS Med. Chem. Lett..

[276] Huang H., Wang W., Xu X., Zhu C., Wang Y., Liu J., Li W., Fu W. (2020). Discovery of 3-((dimethylamino)methyl)-4-hydroxy-4-(3-methoxyphenyl)-N-phenylpiperidine-1-carboxamide as novel potent analgesic. Eur. J. Med. Chem..

[277] Lee H., Ahn S., Ann J., Ha H., Yoo Y.D., Kim Y.H., Hwang J.-Y., Hur K.-H., Jang C.-G., Pearce L.V. (2019). Discovery of dual-acting opioid ligand and TRPV1 antagonists as novel therapeutic agents for pain. Eur. J. Med. Chem..

[278] Benítez-Angeles M., Morales-Lázaro S.L., Juárez-González E., Rosenbaum T. (2020). TRPV1: Structure, Endogenous Agonists, and Mechanisms. Int. J. Mol. Sci..

[279] Déciga-Campos M., Melo-Hernández L.A., Torres-Gómez H., Wünsch B., Schepmann D., González-Trujano M.E., Espinosa-Juárez J., López-Muñoz F.J., Navarrete-Vázquez G. (2020). Design and synthesis of N-(benzylpiperidinyl)-4-fluorobenzamide: A haloperidol analog that reduces neuropathic nociception via σ1 receptor antagonism. Life Sci..

[280] Johnson A.C., Greenwood-Van Meerveld B., Barrett J.E. (2016). Chapter Ten–The Pharmacology of Visceral Pain. Advances in Pharmacology.

[281] Xiong J., Jin J., Gao L., Hao C., Liu X., Liu B.-F., Chen Y., Zhang G. (2020). Piperidine propionamide as a scaffold for potent sigma-1 receptor antagonists and mu opioid receptor agonists for treating neuropathic pain. Eur. J. Med. Chem..

[282] Xiong J., Zhuang T., Ma Y., Xu J., Ye J., Ma R., Zhang S., Liu X., Liu B.-F., Hao C. (2021). Optimization of bifunctional piperidinamide derivatives as σ1R Antagonists/MOR agonists for treating neuropathic pain. Eur. J. Med. Chem..

[283] Kalisha Vali Y., Gundla R., Singh O.V., Tamboli Y., Di Cesare Manelli L., Ghelardini C., Al-Tamimi A.-M.S., Carta F., Angeli A., Supuran C.T. (2019). Spirocyclic sulfonamides with carbonic anhydrase inhibitory and anti-neuropathic pain activity. Bioorganic Chem..

